# Essential Oils and Their Main Chemical Components: The Past 20 Years of Preclinical Studies in Melanoma

**DOI:** 10.3390/cancers12092650

**Published:** 2020-09-16

**Authors:** Marta Di Martile, Stefania Garzoli, Rino Ragno, Donatella Del Bufalo

**Affiliations:** 1 Preclinical Models and New Therapeutic Agents Unit, IRCCS Regina Elena National Cancer Institute, Via Elio Chianesi 53, 00144 Rome, Italy; 2 Department of Chemistry and Technologies of Drugs, Sapienza University, Piazzale Aldo Moro 5, 00185 Rome, Italy; stefania.garzoli@uniroma1.it (S.G.); rino.ragno@uniroma1.it (R.R.); 3 Rome Center for Molecular Design, Department of Drug Chemistry and Technology, Sapienza University, Piazzale Aldo Moro 5, 00185 Rome, Italy

**Keywords:** melanoma, essential oils, angiogenesis, apoptosis, metastasis

## Abstract

**Simple Summary:**

In the last years, targeted therapy and immunotherapy modified the landscape for metastatic melanoma treatment. These therapeutic approaches led to an impressive improvement in patients overall survival. Unfortunately, the emergence of drug resistance and side effects occurring during therapy strongly limit the long-term efficacy of such treatments. Several preclinical studies demonstrate the efficacy of essential oils as antitumoral agents, and clinical trials support their use to reduce side effects emerging during therapy. In this review we have summarized studies describing the molecular mechanism through which essential oils induce in vitro and in vivo cell death in melanoma models. We also pointed to clinical trials investigating the use of essential oils in reducing the side effects experienced by cancer patients or those undergoing anticancer therapy. From this review emerged that further studies are necessary to validate the effectiveness of essential oils for the management of melanoma.

**Abstract:**

The last two decades have seen the development of effective therapies, which have saved the lives of a large number of melanoma patients. However, therapeutic options are still limited for patients without BRAF mutations or in relapse from current treatments, and severe side effects often occur during therapy. Thus, additional insights to improve treatment efficacy with the aim to decrease the likelihood of chemoresistance, as well as reducing side effects of current therapies, are required. Natural products offer great opportunities for the discovery of antineoplastic drugs, and still represent a useful source of novel molecules. Among them, essential oils, representing the volatile fraction of aromatic plants, are always being actively investigated by several research groups and show promising biological activities for their use as complementary or alternative medicine for several diseases, including cancer. In this review, we focused on studies reporting the mechanism through which essential oils exert antitumor action in preclinical wild type or mutant BRAF melanoma models. We also discussed the latest use of essential oils in improving cancer patients’ quality of life. As evidenced by the many studies listed in this review, through their effect on apoptosis and tumor progression-associated properties, essential oils can therefore be considered as potential natural pharmaceutical resources for cancer management.

## 1. Introduction

Melanoma is the third most common cutaneous malignancy and one of the most dangerous forms of skin cancer. It is increasing worldwide and is caused by several factors, including environmental and genetic ones. In primary tumors, the most frequent driver oncogenic mutations (BRAF, NRAS, KIT), as well as mutations responsible for intrinsic resistance, have been identified with genomic analyses. Genetic alterations developed during the acquired resistance and genetic lesions leading to metastasis spreading were also identified [1].

The use of surgery in case of primary tumors is considered curative. With the advent of targeted therapy (MAPK pathway inhibitors) and immunotherapy (immune checkpoint inhibitors), treatment of metastatic melanoma has changed dramatically in recent years. These therapeutic strategies generated unprecedented improvement in patients’ survival [2,3]. Targeted therapy, mostly represented by BRAF inhibitors, shows efficacy in the treatment of BRAF mutated metastatic melanoma when administered as single agents or in combination with MEK inhibitors [4]. Immune checkpoint inhibitors, such as anti-CTLA4 and anti-PD1 monoclonal antibodies, are able to activate the immune response in the host through their ability to unmask the inhibition of the host immune cells [5]. Targeted therapy in combination with immune checkpoint inhibitors has also showed encouraging results in the last few years [6]. Patients who are not eligible candidates for targeted therapy or immunotherapy are treated with systemic chemotherapy, such as dacarbazine, taxanes or temozolomide, while radiation therapy is used for brain metastases or for the treatment of oligometastatic disease [7]. Despite this clinical success, treatment of metastatic melanoma is still ineffective in about half of the treated patients and is often limited by numerous side effects and by the emergence of resistance to both targeted therapy and immunotherapy [8,9,10].

The diagnosis of melanoma and the therapeutic approaches used to contrast the disease, strongly affect the patient’s quality of life. In fact, melanoma patients often experience the risk of disease progression, or even of new primary disease, for several years after diagnosis. Often, anxiety, depression and fear, leading to impaired social functioning, are evidenced in melanoma survivors. For these reasons, there is an urgent need to identify new therapeutic strategies for melanoma patients who do not respond or relapse after therapy, as well as to define new options to improve their quality of life.

In the last two decades, many new anticancer agents have been discovered from natural products as an alternative option to current cancer therapy. This is due to high cost, emergence of drug resistance and the side effects of standard therapies. Several plant-derived drugs, such as camptothecin, vinblastine, vincristine, etoposide, taxol, and paclitaxel have found wide applications in cancer therapy [11,12]. Current cancer research has brought about many promising preclinical results regarding the antiproliferative, anti-inflammatory, antioxidant, antiangiogenic and antimetastatic effect of essential oils (EOs), which are relevant components of aromatic plants. In addition, chemoprevention by EOs could represent a potentially effective option in the fight against cancer and, in particular, against melanoma. At present, EOs are used as a non-invasive therapy with minimal risk intervention that could potentially improve the quality of life of cancer patients and could alleviate the severity of treatment-related liver injuries or, more generally, symptoms in cancer patients undergoing chemotherapy. Alleviation of collateral effects would enhance prognosis status and patients’ survival.

From a survey in scopus [13] with the “essential oil” and “melanoma” keywords, a list of almost 170 references were retrieved and analyzed. Focusing our attention on papers published in the last 20 years, we will summarize and discuss the chemical composition of EOs used in melanoma models, and the molecular mechanism through which EOs and their main components exert an antitumor effect in preclinical melanoma models carrying wild type or mutated BRAF. One section is dedicated to the use of EOs in clinical trials for managing cancer symptoms. This comprehensive summary could be a useful source for a better understanding of EOs’ mechanism of action. It could also help researchers to appreciate and consider the importance of EOs being a potential adjuvant to enhance the efficacy of current available therapy, as well as, for improving patients’ quality of life.

Taking into consideration the studies presented in this review, EOs showing antitumor activity could represent a good opportunity for combination therapy or, particularly, for reducing those side effects caused by current treatments.

## 2. Essential Oils

A wide number of studies have reported that plants contain valuable compounds, including bioactive EOs [14]. EOs are a complex combination of chemical aromatic-smelling low-molecular weight compounds. They are derived from the plants’ secondary metabolism [15], leading predominately to monoterpenes [16], sesquiterpenes [17] and their oxygenated derivatives [18]. They are biosynthesized in different plant organs and parts such as flowers, leaves, fruits and roots [15] and are industrially produced mainly by hydro- or steam-distillation [19,20]. Chemical qualitative and quantitative composition of EOs, which are composed of volatile compounds, is determined using a combination of Gas Chromatography/Flame Ionization Detection, Gas Chromatography/Mass Spectrometry and determination of their Kovats index [21] and/or Linear Retention Indices [22]. The chemical composition of aromatic plants furnishing EOs active against melanoma models is reported in Table 1. Common and family names of the plants, as well as the percentage of the main components identified in each EO, are also included.

Anticancer activities of EO mixtures as a whole and only a hypothetical correlation between the chemical profile and the anticancer activity have been described for some EOs. In some cases, EOs’ bioactive properties were related to the anticancer activity of specific components. Due to EOs’ complex chemical composition, the additive, synergistic or anti-synergistic roles of individual EO constituents are currently being investigated to establish the possible pharmacological activity thereof [92,93]. This categorization is even more complicated due to the ‘chemotype’ concept, in which the same plant could produce different EOs characterized by different chemical composition profiles and, hence, different biological properties [94]. *Ocimum tenuiflorum* (holy basil), *Thymus vulgaris* (thyme), *Lavandula angustifolia* (lavender) and *Mentha piperita* (peppermint) are examples of plants with several chemotypes [95]. Despite this mix up, an effort to characterize EOs is currently taking place in medical and pharmaceutical fields. This characterization could help to obtain a clearer indication of EOs’ uses in traditional medicine, chemical or pharmaceutical, as witnessed by almost 5000 articles published in PubMed (http://pubmed.ncbi.nlm.gov/). In the last decade, an average positive increment of more than 7% per year was observed in this field [96].

When referring to EOs, their chemical composition and biological activities strictly depend on habitat, climate condition, season, agronomic practices, soil type, extraction procedures, as well as the harvesting stages and storage conditions of plants [31,36,37,51,75,97,98,99,100,101,102,103]. All these elements should be taken into account. By analyzing gene expression patterns and metabolic fingerprints, recently Spring’s group identified environmental factors as regulatory factors of biosynthetic pathways [104]. Substantial variability was also reported according to the part of plant used for extraction of EOs [83]. Examples are EOs from *Helichrysum microphyllum* [45] and from *Liriodendron tulipifera*, and their main components β-elemene and (E)-nerolidol, showing antiproliferative activity in human melanoma cells strictly depending on harvesting period [51]. Moreover, EOs from *Chrysanthemum boreale* Makino, showed different levels of their component contents and bioactivities among the harvesting stages [31], while phytoconstituents and bioactivities of EOs from *Curcuma kwangsiensis*, strictly depended on the natural habitat [37]. Another example is provided by chemotaxonomical analysis of *Artemisia absinthium*, *Salvia officinalis, Tanacetum vulgare* and *Thuja occidentalis*, the amount of thujones (α-thujone and β-thujone) present in the EOs of the four species being strictly related to the plant organ and to its developmental phase [105]. Moreover, exposure to ultraviolet (UV) light was reported to induce deterioration of EOs’ biochemical profiles [49] or activation of some EOs [34]. In this regard, the antiproliferative effect of both *Citrus medica* and *Citrus bergamia* EOs and their constituent bergapten, was observed in human melanoma cells after exposure to UV irradiation, thus indicating UV irradiation’s ability to activate EOs [34]. In some cases, derivatives of EOs are designed to increase EOs’ half-life. This is the case of farnesyl-O-acetylhydroquinone, geranyl-O-acetylhydroquinone, geranyl ester and farnesyl ester derived from geraniol and farnesol. Structure–activity relationship studies reported the ability of these derivatives to reduce proliferation of mouse melanoma cells more efficiently than the parental compounds [106,107]. Furthermore, 6-(menthoxybutyryl)thymoquinone, the terpene conjugate derivative of thymoquinone, was shown to be more active in human melanoma cells than its parental compound [108]. A cumulative impact of some EOs’ components was also evidenced: farnesol and nerolidol in combination showed an enhanced antiproliferative effect in mouse melanoma cells when compared to exposure to single treatments [109].

For all their biological effects, EOs can be considered as an interesting source for therapeutic, food preservation and/or nutraceutical uses [110,111,112,113,114].

## 3. Mechanism of Action of EOs in Melanoma

EOs and some of their components exert antitumor activity in melanoma models by affecting multiple pathways, including inhibition of in vitro cell proliferation, alteration of cell distribution in the different cell cycle phases, induction of apoptosis, inhibition of in vitro cell invasion and migration, in vivo tumor growth and metastasization and in vitro/in vivo angiogenesis. Several EOs also act as chemopreventive agents in melanoma, reduce melanogenesis and show antioxidant properties.

The most frequently used human melanoma models are represented by M14, A2058, A375 cells and SK-MEL variants. Murine melanoma B16 cells, that originate in the syngeneic C57BL/6 (H-2b) mouse strain, and its derivatives B16-F1, B16-F10, B16-F10-Nex2, B16-Bl6, B164A5 [115,116,117,118] represent the most used in vivo models. They are employed to evaluate the effect of EOs on tumor growth and metastasization, as well as tumor angiogenesis, after subcutaneous or intravenous (lateral tail vein) cell injection [119]. In some cases subretinal, intradermal, intracerebral injection of melanoma cells is employed [38,86,120,121]. Routes of EO administration include oral, intraperitoneal, intravitreal, peritumoral, topical as well as inhalation (fragrant environmental).

### 3.1. Inhibition of Cell Proliferation

Cell viability and proliferation can be detected by a wide range of assays based on several cell functions, including mitochondrial enzyme and cellular uptake activity, cell membrane permeability and ATP production. The most used assay is a colorimetric test that evaluates the reduction of yellow 3-(4,5-dimethythiazol-2-yl)-2,5-diphenyl tetrazolium bromide by mitochondrial succinate dehydrogenase [122]. Analysis of cell proliferation can also be performed by alamarBlue and PrestoBlue assays, both using reduction of resazurin as an indicator of cell viability [122]. Detection of 5-bromo-2′-deoxyuridine (BrdU)- or 5-ethynyl-2′-deoxyuridine (EdU)- labeled DNA also represents a valid method to detect viable cells: BrdU or EdU are efficiently incorporated into DNA of replicating cells by substituting thymidine and their binding to DNA can be detected with specific antibodies [123]. Moreover, the thymidine incorporation assay, based on measuring the incorporation of methyl-[3H]-thymidine into the DNA of dividing cancer cells, is frequently used to estimate cell proliferation [124].

A high number of EOs and their components have been found to reduce in vitro proliferation/viability of melanoma cells, and in some cases the cytotoxic potential has been predicted by in silico studies [125].

EOs and their main components demonstrated to reduce in vitro proliferation of melanoma cells are reported in Table 2. The used melanoma models are also indicated in the Table.

### 3.2. Alteration of Cell Cycle Distribution

Cell cycle transition is a process involving multiple checkpoints, which control growth signals, cell size and DNA integrity. It is regulated by active forms of cyclin-dependent kinases, which control the passage of cells from one phase of the cell cycle to another. Cyclin-dependent kinases act as cell cycle regulators to elicit cell cycle arrest in response to DNA damage [153]. Cytofluorimetric analysis of cells stained with the DNA intercalator, propidium iodide, represents the most used methods to analyze cell cycle transition.

Several authors demonstrated the ability of EOs or their constituents to induce DNA damage in melanoma cells, and consequently inducing delay/arrest in the different cell cycle phases. Recently, Ramadam et al. reported the *Melaleuca alternifolia* EO (Tea tree oil, TTO) ability to induce cell cycle arrest at the G2/M phase of A375 cells [129], while in a previously published study, Beilharz’s group described the TTO ability to elicit G1 cell cycle arrest in B16 cells [130]. In addition, α-santalol, the main component of *Santalum album* (Sandalwood oil) [154], in UACC-62 cells induced G2/M phase arrest through down-regulation of proteins critical for G2/M transition, such as cyclin A/Cdk2 and cyclin B/Cdc2 complexes, as well as microtubule depolymerisation. It also increased expression of wild-type p53 [155]. Eugenol, a component present in many EOs including *Syzygium aromaticum* (Clove oil) and *Cinnamomum zeylanicum* [156], abrogated the G2/M phase in A2058 but not in SK-MEL-28 cells. It was also reported to arrest WM1205Lu cells in the S phase of the cell cycle through the inhibition of E2F1 transcription factor activity. E2F1 is a key cell cycle regulator, targeting genes that encode proteins involved in G1/S transition [134,135]. Eugenol also reduced the expression of proliferation cell nuclear antigen in A2058 cells [134]. Given the role of E2F1 in melanoma progression and resistance to therapy [157,158], the authors also suggested that eugenol could be developed as an E2F-targeted agent for melanoma treatment. Farnesol (a mixture of trans, trans and cis, trans isomers) and nerolidol, present in different EOs including that from *Psidium guajava* [159], induced an increase in cells in the G0/G1 phase, concomitant with a reduction in the S phase, and a cumulative impact of the two compounds was evidenced in B16 cells [109].

The different response between cell lines sometimes observed after exposure to the same EO indicate a non-generalizable and cell-type specific effect or is due to differences in the composition of the EOs’ used in the distinct studies. The genetic background of the cell lines tested should be also considered: some melanoma cells harbor activating BRAF^V600E^ mutations (A375, M14, A2058, SK-MEL-5, SK-MEL-19, SK-MEL-28, UACC-62, UACC-257, 518A2, G-361, WM266) not present in other ones (B16, Sbcl2, CHL1, WM3211, SK-MEL-147, MeWo, RPMI-7932) (https://web.expasy.org/cellosaurus/), or harbor different mutations (i.e., NRAS, CDKN2A, TP53, TERT). A better understanding of these cell-to-cell differences is crucial for a deeper comprehension of the EO mechanism of action.

EOs and their main components demonstrated to induce cell cycle perturbation of melanoma cells are reported in Table 3. The used melanoma models are also listed in the Table.

### 3.3. Induction of Apoptosis

Apoptosis is a programmed cell death characterized by the activation of a group of intracellular caspases leading to a cascade of events connected with various substrates, including poly(ADP-ribose) polymerase-I (PARP), and internalization by phagocytes [163,164]. Hallmarks of apoptosis include cell shrinkage, formation of apoptotic bodies, DNA fragmentation, heterochromatin aggregation and activation of caspases and substrates such as PARP. Exposure of phosphatidyl serine on the cell surface, change in the mitochondrial membrane potential, as well as change in the ratio of mRNA expression of pro- and anti-apoptotic proteins, also represent features of apoptotic cells. The presence of a sub-G0/G1 population in the cell cycle is considered indicative of DNA damage and apoptosis [165]. Comet assay, a genotoxic test, can be used as an indicator of early apoptosis, since cells entering apoptosis undergo DNA fragmentation resulting in the characteristic images. In these images the tail and the head indicate, respectively, fragmented and intact DNA [166].

Most EOs have been reported to cause cell death of melanoma cells, primarily inducing apoptosis, and hallmarks of apoptosis were recognized in melanoma cells treated with a huge number of EOs or their components. TTO induced apoptosis in A375 cells through the activation of caspases 3, 7 and 9, upregulation of p53 and Bax proapoptotic proteins and downregulation of bcl-2 antiapoptotic protein [129]. TTO and its main active component, terpinen-4-ol, also induced apoptosis in both adriamycin-sensitive and -resistant M14 cells: interaction with plasma membrane and subsequent reorganization of membrane lipid architecture has been identified as a possible mechanism through which TTO induced caspases dependent apoptosis [52,167]. Moreover, EO from *Salvia verbenaca* showed proapoptotic effects in M14 cells, and the EOs from cultivated plants exhibited major effects when compared to those growing in natural sites [75], thus further confirming the relevance of cultivar conditions on EOs activity. The same group also pointed a proapoptotic activity of *Salvia officinalis* EO in A375, M14 and A2058 cells, the percentages of main components depending on environmental factors [101]. The authors also suggested activation of apoptosis by *Salvia rubifolia* and *Salvia bracteata* EOs in M14 cells using a Comet assay [72,166]. *Boswellia carterii* EO (frankincense oil) was found to induce apoptosis through downregulation of Bcl-2 and up regulation of Bax proteins in B16-F10 cells, while inducing down regulation of Mcl-1 and cleavage of caspase 3 and 9 and PARP in human melanoma FM94 cells. On the contrary, proliferation of normal human epithelial melanocytes was not affected [146]. EOs extracted from *Pituranthos tortuosus* and *Annona Vepretorum* and their major constituents spathulenol, o-cymene and α-pinene, induced apoptosis of B16-F10 cells [25,66].

Hallmarks of apoptosis were also recognized in cells treated with several EO components, including camphene, α-pinene, eugenol, linalool, zerumbone, carvacrol, thujone, curzerene, citral, thymoquinone, isoegomaketone and menthol. Camphene isolated from *Piper cernuum* EO, induced apoptosis in B16-F10-Nex2 cells through loss of mitochondrial membrane potential, activation of caspases 3, endoplasmic reticulum stress, release of calcium, increased expression of high mobility group box 1 (HMGB1) and cell surface calreticulin [62]. The induction of HMGB1 and calreticulin after treatment with camphene could elicit immunogenic cell death, a relevant pathway for the activation of the immune system [168]. In B16-F10-Nex2 cells, α-pinene, a component present in many EOs including those from *Schinus terebinthifolius, Tridax procumbens, Pituranthos tortuosus, Annona Vepretorum* and *Boswellia carterii* [169], induced disruption of the mitochondrial potential, production of reactive oxygen species (ROS), activation of caspase 3, aggregation of heterochromatin, fragmentation of DNA and exposure of phosphatidyl serine on the cell surface [149,169]. Experiments performed to identify the structure/activity relationship, indicated the presence of a double bond in the α-pinene structure as crucial for its cytotoxic potential against both B16-F10-Nex2 and A2058 cells [78].

Proapoptotic properties, in terms of DNA fragmentation, phosphatidylserine exposure, and mitochondrial damage were reported by eugenol and the 6,6′-dibromo-dehydrodieugenol (S) enantiomeric form, in established and primary melanoma cells from patient tissue samples, with no effect on fibroblasts. Clastogenesis analysis and clonogenic assay also pointed out the ability of eugenol, respectively, to induce DNA breaks and to reduce colony forming potential, through a direct cytotoxicity or a lingering antiproliferative effect, in A2058 and SK-MEL-28 cells [134,136,170]. Scanning electron microscopy and transmission electron microscopy demonstrated that linalool, present in several EOs including those from *Citrus bergamia*, induced morphological changes and apoptosis in RPMI-7932 human melanoma cells while not affecting proliferation of normal keratinocytes [160].

The proapoptotic effect of zerumbone, one of the main constituents of EOs from *Zingiber zerumbet* and *Cheilocostus speciosus* [171] was proved in human melanoma CHL-1 cells through induction of ROS, reduction of mitochondrial matrix potential and mitochondrial biogenesis mediated by reduced mitochondrial ATP synthesis, mitochondrial DNA levels, and mRNA expression of mitochondrial transcription factor A level, a mitochondrial biogenesis factor [138]. Activation of apoptosis through downregulation of Bcl-2 and upregulation of Bax and cytochrome c gene and protein levels, as well as activation of caspases 3, was also observed in A375 cells after treatment with zerumbone [139]. Morphological and biochemical features of apoptosis in A375 cells were also induced by carvacrol, the main constituent isolated from *Coleus aromaticus* and present in other EOs, such as those from *Origanum ehrenbergii, Origanum syriacum* and *Satureja hortensis* [127]. EO fractions rich in thujone, isolated from *Thuja occidentalis* [172], induced apoptosis in A375 cells, with minimal growth inhibitory responses when exposed to normal cells [131]. Curzerene from *Eugenia uniflora* activated apoptosis in SK-MEL-19 cells [43].

Induction of apoptosis, necrosis, and autophagy was observed in B16-F10 cells after treatment with citral, a key component of EOs from *Cymbopogon citrates*, *Melissa officinalis* and *Verbena officinalis*. Apoptosis was associated with reduction of extracellular signal-regulated kinase-1 (ERK-1) and -2 (ERK-2), AKT and nuclear factor kappa B (NF-kB), as well as, induction of oxidative stress, DNA lesions, ROS and lipid peroxidation. SK-MEL-147 and UACC-257 cells showed a lower sensitivity to citral, when compared to B16-F10 cells [133].

Data pointing toward a proapoptotic activity of thymoquinone, a constituent of the EOs from *Nigella sativa* and *Thymus species,* have been also reported in B16-F10 cells through JAK2/STAT signal transduction [121]. Isoegomaketone from *Perilla frutescens* trigged ROS-mediated caspase-dependent and -independent apoptosis in B16 cells. In vitro studies were supported by in vivo experiments demonstrating that oral gavage of isoegomaketone in mice subcutaneously carrying B16 melanoma inhibited tumor growth, induced apoptosis, as well as increased Bax/Bcl-2 ratio [60].

Menthol, a compound present in EOs such as peppermint and mint has been reported to exert in vitro cytotoxic effect in A375 cells and to induce morphological changes, such as cell shrinkage and ruptured membranes, indicative of apoptosis. Decrease in transient receptor potential melastatin 8 (TRPM8), at the transcript level, was also evidenced following treatment with menthol. TRPM8 is a membrane receptor involved in the regulation of calcium ion influx and melanocytic behavior, and upregulated in melanoma [173]. The authors also hypothesized that the effect of menthol on TRPM8 expression could be linked to both decrease in cell proliferation and increase in cell death [137,161]. Menthol induced cytotoxicity was also pointed out in G-361 melanoma cells through a TRPM8-dependent mechanism only when using high doses of menthol [162], thus indicating a significant difference between A375 and G-361 cells in the sensitivity to menthol.

EOs and their main components demonstrated to induce apoptosis in melanoma cells are reported in Table 3. The in vitro and in vivo melanoma models used are also listed in the Table.

### 3.4. Induction of Necrosis or Modulation of Autophagy

Necrosis is a non-programmed cell death that, contrary to apoptosis, does not use a highly regulated intracellular program. Necrotic cells have usually lost cell membrane integrity and release products and enzymes in the extracellular space, with consequent activation of an inflammatory response. They are taken up and internalized by macropinocytotic mechanisms [163].

Autophagy is a recycling process playing a relevant role in cell survival and maintenance. Through the analysis of LC3II protein expression, presence of dot-like formations of endogenous LC3 protein and its colocalization with the lysosome marker LAMP-1, degradation of the specific autophagy substrate p62, use of early and late autophagy inhibitors, it is possible to analyze whether a particular compound affects autophagy, inducing autophagy rather than decreasing autophagosomal turnover [174,175].

Only a few EOs have been reported to induce necrosis in melanoma cells. Among them, TTO and its major active component, terpinen-4-ol, induced necrotic cell death coupled with low level apoptotic cell death in B16 cells. Necrosis was evidenced by ultrastructural features, including cell and organelle swelling, identified by video time lapse microscopy and transmission electron microscopy [130]. Measurement of lactate dehydrogenase (LDH) release from the cytosol into the supernatant demonstrated cellular membrane damage associated with necrosis in B16-F10 cells exposed to citral. Apoptosis induction and signs of autophagy were also evident after treatment with citral [133]. EOs distilled from *Salvia bracteata, Salvia rubifolia, Salvia aurea, Salvia judaica and Salvia viscosa* induced apoptosis and necrosis in M14 cells [71,72].

Regarding autophagy, some EOs or their components, have been found to affect basal autophagy or to trigger autophagy induced by serum starvation and/or rapamycin in cancer cells from different origin, indicating an mTOR independent mechanism [176]. α-Thujone, D-limonene, terpinel-4-ol and β-elemene are all components of EOs reported to affect in vitro and/or in vivo melanoma growth or melanogenesis [120,131,144,152,177] and to induce autophagy, respectively, in glioblastoma [178], neuroblastoma [179], leukemic [180] and breast carcinoma [181] cells. To the best of our knowledge, no studies exist regarding the ability of these or other EO components to induce autophagy or to affect autophagic flux in melanoma cells. Generation of autophagic vacuoles in B16-F10 cells was observed after treatment with citral. The authors suggested that B16-F10 cells shifted cellular metabolism, trying to recycle damaged structures by oxidative stress under treatment with citral, but no further characterization of autophagy was provided [133].

EOs and their main components demonstrated to induce necrosis or to modulate autophagy in melanoma cells are reported in Table 3. The in vitro melanoma models used are also listed in the Table.

### 3.5. Inhibition of Angiogenesis and Lymphangiogenesis

Angiogenesis, the formation of new blood vessels from existing microvessels, plays a relevant role in the growth and metastasization of many tumors, including melanoma [182]. Lesions at the beginning stage do not grow in the absence of angiogenesis or inflammation [183]. Furthermore, vasculogenic mimicry (VM) and lymphangiogenesis contribute to the metastatic spread of melanoma [184]. VM and lymphangiogenesis represent, respectively, the ability of cells to form networks of vessel-like channels and the formation of lymphatic vessels from pre-existing ones. Thus, inhibition of angiogenesis, VM or lymphangiogenesis, could represent a valid strategy for melanoma prevention and treatment. Vascular endothelial growth factor (VEGF) is one of the most important angiogenic factors that, through its binding to the receptor tyrosine kinase VEGFR, and the formation of a VEGF–VEGFR complex, induces angiogenesis (VEGF-A) or lymphangiogenesis (VEGF-C, VEGF-D) [185,186].

**Figure 1:**
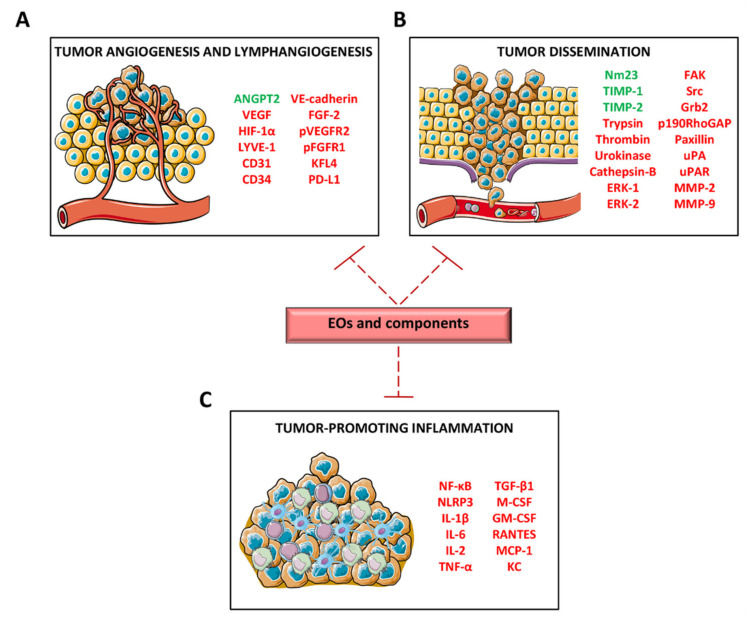
EOs and their components reduce tumor angiogenesis, lymphangiogenesis (**A**) and tumor metastasization, by targeting proteins responsible for tumor dissemination (**B**) and tumor-promoting inflammation (**C**). Angiopoietin 2 (ANGPT2), vascular endothelial growth factor (VEGF), hypoxia responsive factor 1α (HIF-1α), lymphatic vessel endothelial receptor (LYVE-1), cluster of differentiation 31 (CD31), cluster of differentiation 34 (CD34), vascular endothelial cadherin (VE-cadherin), fibroblast growth factor 2 (FGF-2), phospho vascular endothelial growth factor receptor 2 (pVEGFR2), phospho fibroblast growth factor receptor-1 (pFGFR1), krüppel-like factor 4 (KFL4), programmed death-ligand 1 (PD-L1), tissue inhibitor of metalloproteinase-1 (TIMP-1) and -2 (TIMP-2), extracellular signal-regulated kinase-1 (ERK-1) and -2 (ERK-2), focal adhesion kinase (FAK), growth factor receptor-bound protein 2 (Grb2), urokinase-type plasminogen activator (uPA), uPA receptor (uPAR), metalloproteinases-2 (MMP-2) and -9 (MMP-9), nuclear factor kappa B (NF-kB), NLR family pyrin domain containing 3 (NLRP3), interleukin 1β (IL-1 β), interleukin 6 (IL-6), interleukin 2 (IL-2), tumor necrosis factor-α (TNF-α), transforming growth factor β1 (TGF-β1), macrophage colony stimulating factor (M-CSF), granulocyte-macrophage colony stimulating factor (GM-CSF), monocyte chemoattractant protein-1 (MCP-1), keratinocyte chemoattractant (KC). Proteins that are upregulated by EOs or their components are reported in green, proteins that are downregulated by EOs or their components are reported in red. Parts of the figure are drawn using pictures from Servier Medical Art (https://smart.servier.com).

The ability to reduce angiogenesis has been highlighted in several EOs and their components. In particular, in vitro endothelial cells and in vivo/ex vivo assays such as, rat aortic ring, matrigel plug and chick embryo chorioallantoic membrane (CAM), have been used to study the effect of EOs on the formation of new blood vessels from pre-existing ones. Figure 1A summarizes pro- and anti- angiogenic factors regulated by EOs or their components.

EO from *Pistacia lentiscus* (Mastic oil) has been extensively studied due to its antiangiogenic effect attributed to both its activities on in vitro endothelial cell proliferation and in vivo microvessel formation. It also inhibited VEGF release by B16 cells. Investigation of underlying mechanism by *Pistacia lentiscus* EOs in endothelial cells demonstrated activation of RhoA, a regulator of neovessel organization [64]. *Tridax procumbens* EO, when administered intraperitoneally, inhibited capillary formation in B16-F10 injected intradermally on the shaven ventral skin of C57BL/6 mice [86]. Using the same experimental model, the *Plectranthus amboinicus* EO ability to reduce tumor-directed blood vessel formation was also evidenced [67]. In addition, EO from *Curcuma zedoaria* was reported to suppress in vitro proliferation of human umbilical vein endothelial cells, sprouting vessels of aortic ring and formation of microvessels in CAM. The antiangiogenic effect was also confirmed by immunohistochemical analysis of tumors showing a reduced expression of the endothelial marker CD34 after oral administration of *Curcuma zedoaria* EO in C57BL/6 mice carrying B16-Bl6 melanoma [38].

Regarding EO components, several groups reported that β-elemene, one of the most active constituents of *Curcuma zedoaria* and *Curcuma wenyujin* EOs, inhibited the VEGF-induced sprouting vessel of rat aortic ring, microvessel formation of CAM, as well as CD34 and VEGF expression in C57BL/6 mice carrying B16-F10 melanoma after subretinal injection. VEGF expression in serum and lung of mice was also inhibited following treatment with β-elemene [120,152]. In vitro and in vivo studies carried out by Jung’s group highlighted the ability of dietary β-caryophyllene, a component found in many EOs (basil, black pepper, cinnamon, cannabis, lavender, rosemary, cloves, oregano), to suppress high fat diet (HDF)-induced in vitro and in vivo angiogenesis and lymphangiogenesis. In particular, dietary β-caryophyllene reduced the expression at transcriptional and protein level of hypoxia inducible factors 1α, VEGF-A, CD31 and VE-cadherin observed in the tumors of HFD-fed mice. In addition, the HFD-stimulated expression of VEGF-C, VEGF-D, VEGF-R3 and lymphatic vessel endothelial receptor was prevented by β-caryophyllene supplementation in the diet. The authors indicate the effect of β-caryophyllene in angiogenesis as one of the most important mechanisms for reduced tumor growth in β-caryophyllene-fed mice [140].

Even if a direct effect on melanoma angiogenesis has not yet been reported, several EOs or their components have been found to affect in vitro endothelial functions and/or in vivo neovascularisation. Among them, EOs from *Hypericum perforatum* [187] and *Myristica fragrans* [56] should be mentioned, together with coated-dacarbazine eugenol liposomes, for their ability to reduce proliferation and migration of endothelial-like cells [188]. *Citrus lemon* EO nanoemulsions have been found to decrease angiogenesis in CAM [189]. Interestingly, when analyzing the effect of the ointment prepared from *Salvia officinalis* EO on the healing process of an infected wound mouse model, Farahpour et al. demonstrated that, in addition to antioxidant and anti-inflammatory properties, *Salvia officinalis* EO accelerates the wound healing process. This process requires coordination of overlapping distinct cellular activities including angiogenesis. Promotion of the healing process by *Salvia officinalis* EO was attributed to enhanced angiogenesis through upregulation of VEGF and fibroblast growth factor-2 (FGF-2) expression. An increase in the number of blood vessels and fibroblasts, through cyclin-D1 pathway activation and enhanced expression of Bcl-2, was also observed [190]. A positive effect on wound healing process has also been described for other EOs such as lavender EO, TTO, *Alpinia zerumbet* and *Chrysantemum boreale* Makino EO and their use has been suggested for the treatment of wounds, burns, abscesses or diseases such as diabetes [191,192,193,194]. Thus, the antiangiogenic property or the positive effect on wound healing showed by some EOs should be considered when proposing them for treatment of cancer or other diseases.

The ability to interfere with the process of angiogenesis has also been attributed to zerumbone, perillyl alcohol and curcumol, three components of many EOs. Zerumbone, has been found to inhibit proliferation, migration, and morphogenesis, but not viability of endothelial cells, as well as the outgrowth of new blood vessels in rat aortic rings, vessel formation in the matrigel plug and CAM assays. All these effects were mediated by downregulation of phosphorylation of VEGFR2 and fibroblast growth factor receptor-1, two essential signaling pathways for angiogenesis [195,196]. Perillyl alcohol, a component of *Anethum graveolens, Conyza newii* and *Citrus limon* EOs [197], blocked the growth of new blood vessel in the in vivo CAM assay and inhibited the in vitro morphogenic differentiation of endothelial cells. Perillyl alcohol also reduced proliferation and induced both apoptosis and the expression of the antiangiogenic molecule, angiopoietin 2, in endothelial cells, indicating that it exerted its effect through both vessel regression and neovascularization suppression [198]. Curcumol, a representative component for the quality control of the EO of *Curcuma wenyujin*, inhibited angiogenesis by reducing PD-L1 expression in endothelial cells. In particular, addition of curcumol reduced the expression of VEGF and metalloproteinase-9 (MMP-9) and tube formation induced by PD-L1. It also cooperated with the ability of PD-L1 silencing to downregulate VEGF and MMP-9 expression and morphogenesis of endothelial cells [199].

EOs and their main components demonstrated to affect angiogenesis by using both endothelial and melanoma models are reported in Table 4. The used in vitro and in vivo models are also listed.

### 3.6. Alteration of In Vitro Tumor Progression-Associated Functions and Inhibition of In Vivo Tumor Growth and Metastasization

Tumor metastasization, the spread of tumor cells from the primary site to distant organs, represents the main cause of death of cancer patients, including those affected by melanoma. Thus, new therapeutic approaches, which are able to block functions associated to tumor progression, or even to prevent metastasization, represent a big turning point for cancer therapy. Several studies reported that the antimetastatic potential of EOs goes across the regulation of inflammatory cytokines and chemokines. Figure 1B,C summarizes factors regulated by EOs and responsible for tumor dissemination and tumor-promoting inflammation.

Several studies demonstrated EOs’ ability to affect in vitro tumor progression-associated functions and in vivo tumor growth and metastasization. EO from *Alpinia zerumbet* was shown to inhibit transforming growth factor (TGF)-β1-induced endothelial-to-mesenchymal transition in endothelial cells through regulation of Krüppel-like factor 4. Activation of endothelial-to-mesenchymal transition, a process in which endothelial cells switch from the endothelial to a mesenchymal-like phenotype, cell marker and functions, contribute to cancer progression [141].

Treatment of A375 cells with EOs from *Satureja hortensis* inhibited the in vitro cell migration process, while not affecting migration of normal keratinocytes and fibroblasts [77]. TTO and its main active component, terpinen-4-ol, interfered with in vitro cell migration and invasion of adriamycin-sensitive and -resistant M14 cells by inhibiting the intracellular pathway induced by the multidrug transporter p170 glycoprotein [142]. In vivo results also reported that topical TTO formulation was able to slow the growth of B16-F10 melanoma subcutaneously injected in C57BL/6J mice. The treatment was accompanied by a quick and complete disappearance of skin irritation together with recruitment of neutrophils and other immune effector cells in the treated area, while it did not induce systemic toxicity [147]. Both studies highlighted the potential of TTO in topical formulations as a promising chemopreventive candidate or as an alternative topical antitumor treatment against melanoma.

Through its component β-ursolic acid, *Salvia officinalis* EO, inhibited proteases implicated in the mechanisms by which tumor cells metastasize, such as serine proteases (trypsin, thrombin and urokinase) and the cysteine protease cathepsin B. In vivo inhibition of lung colonization of B16 mouse melanoma cells by intraperitoneal administration of β-ursolic acid was also highlighted [148]. After oral administration, *Curcuma zedoaria* EO was reported to suppress in vivo growth of B16-Bl6 tumors after subcutaneous cell injection into the left oxter of C57BL/6 mice, and their metastasization to the lung. A reduced expression of MMP-2 and MMP-9 in serum of treated mice was also evidenced after treatment with *Curcuma zedoaria* EO [38].

Intraperitoneal treatment with *Boswellia carterii* EO reduced the tumor burden in C57BL/6 mice carrying the B16-F10 model, while it did not elicit a detrimental effect on body weight. The authors also reported hepatoprotection by the EO [146]. Considering that liver injury is a frequent consequence of melanoma drug treatments [200,201], this represents an important remark. In the same experimental model, EO from *Pituranthos tortuosus* inhibited in vitro cell migration and invasion, focal adhesion and invadopodia formation. It also induced downregulation of kinases, and molecules involved in cell movement and migration, such as FAK, Src, ERK, p130Cas and paxillin. A decreased expression of p190RhoGAP and Grb2, which impaired cell migration and actin assembly, was also induced by the *Pituranthos tortuosus* EO. In vivo treatment of B16-F10 carrying mice with intraperitoneal administered *Pituranthos tortuosus* EO led to impaired tumor growth with no sign of abnormal behavior or adverse toxicity [66].

*Zornia brasiliensis* [91] and *Annona vepretorum* [25] EOs intraperitoneally administered in C57BL/6 mice subcutaneously carrying B16-F10 melanoma elicited antitumor activity. Importantly, microencapsulation of the *Annona vepretorum* EO with β-cyclodextrin, used to form inclusion complexes with EO and to improve their characteristics, further increased in vivo tumor growth inhibition with respect to free-EO to the level induced by dacarbazine [202]. While not showing any lethal effect/abnormality on mice when injected intraperitoneally, EO from *Tridax procumbens* elicited a reduction of tumor lung nodules of B16-F10 cells injected through the tail vein. Increased apoptosis, associated with enhanced p53 expression, was also observed after treatment. Decrease in body weight, increase in white blood cells and decrease in haemoglobin observed in untreated group, were almost normalized in the EO treated group [86]. Using the same experimental model, the same group also evidenced the ability of EO from *Plectranthus amboinicus* to decrease experimental metastase formation [67].

Moreover, some EO components were reported to inhibit tumor progression-associated properties and in vivo metastasization. In this regard, zerumbone [138] and curzerene from *Eugenia uniflora* EO [43] inhibited in vitro migration of CHL-1 and SK-MEL-19 cells, respectively.

In the Section 3.5, we reported that β-caryophyllene reduced angiogenesis and lymphangiogenesis. In addition to these effects, β-caryophyllene in tumor tissues also reduced M2 macrophages and macrophage mannose receptors. Reduction of cytokines promoting macrophage recruitment and differentiation toward M2 type, such as keratinocyte chemoattractant, monocyte chemoattractant protein-1 (MCP-1) and macrophage colony stimulating factor, were also observed. β-Caryophyllene also increased the number of apoptotic cells and the expression of apoptosis related proteins Bax and activated caspases 3. In the adipose tissues surrounding the lymphnode, β-caryophyllene reduced M2 macrophages and blocked the CCL19-CCL21/CCR7 axis, a signaling pathway important for recruitment of CCR7-expressing cancer cells or leukocytes to lymphnodes. The authors suggested the use of β-caryophyllene for people with high risk of melanoma and/or consuming a high-fat diet regimen [140].

The antimetastatic potential of thujone was demonstrated after injection of B16-F10 cells in the lateral tail vein of C57BL/6 mice. In addition to the reduction of lung nodules, thujone administration also reduced expression of MMP-2, MMP-9, ERK-1, ERK-2, and VEGF and upregulated the expression of nm-23, tissue inhibitor of metalloproteinase-1 (TIMP-1), and TIMP-2 in the lung tissues and the production of pro-inflammatory cytokines such as tumor necrosis factor-α (TNF-α), interleukin (IL)-1β, IL-6, IL-2 and granulocyte-monocyte colony-stimulating factor. In the same model, thujone also inhibited in vitro secretion of MMP-2 and MMP-9 and the adhesion of tumor cells to the collagen-coated plate, as well as cell invasion and migration [144].

Intraperitoneally administered β-elemene was reported to inhibit in vivo growth and metastasization of C57BL/6 mice carrying B16-F10 melanoma through downregulation of tumor promoting factors such as MMP-2, MMP-9, VEGF, urokinase-type plasminogen activator (uPA) and uPA receptor. Reduction of melanin content in lung confirmed the antimetastatic effect of β-elemene [120,152]. The ability of intravitreal administered β-elemene to block the growth of subretinal injected B16-F10 cells in C57BL/6J mice was also reported [120].

Intraperitoneal administration of limonene and perillic acid remarkably reduced the experimental metastatic tumor nodule formation of C57BL/6 mice intravenously injected with B16-F10 cells and increased the life span of animals. Limonene and perillic acid treatment also induced an increased expression in lung tissues and an enhanced serum content of sialic acid and uronic acid, two biochemical markers playing important roles in tumor growth and metastasis, including cell–cell communication and tumor cell escape from immune surveillance [150,203,204]. An antimetastatic effect by α-pinene from *Schinus terebinthifolius* Raddi was reported when B16-F10-Nex2 cells were injected intravenously and C57BL/6 mice treated intraperitoneally [149]. Recent findings provided important insights into the mechanism through which α-pinene induced tumor regression in melanoma models. Some authors supposed the relevance of environment in minimizing cancer growth and reported that α-pinene has no inhibitory effect on melanoma cell proliferation in vitro, but indicated activation in the hypothalamus/sympathetic nerve/leptin axis tumor growth (decreased plasma leptin concentration) and in the immune system (increased the numbers of B cells, CD4+ T cells, CD8+ T cells, and NK cells) as possible mechanisms through which exposure to a fragrant environment containing α-pinene suppressed B16 tumor growth in C57BL/6 mice [205,206].

Camphene derived from *Piper cernuum* EO, when injected peritumorally, exerted antitumor activity in vivo by inhibiting subcutaneous growth of B16-F10-Nex2 in C57BL/6 mice, while it did not induce toxic effects, weight loss, or behavior alterations in mice [62]. Oral administration of thymoquinone reduced experimental metastases of B16-F10 model through destabilization of the oncogene MUC4 mRNA by tristetraprolin, a RNA binding protein regulating the MUC4 transcript [151], while intraperitoneal administration inhibited the growth of the B16-F10 intracerebral model and increased the median overall survival of C57BL/6J mice. Reduction by thymoquinone of inflammatory cytokines, such as MCP-1, TGF-β1, and RANTES, as well as induction of apoptosis, were identified as possible causes of tumor inhibition [121]. Another study evidenced thymoquinone’s ability to inhibit the in vitro migration of both human (A375) and mouse (B16-F10) melanoma cells and to suppress B16-F10 metastasis in C57BL/6 mice by inhibition of NLRP3 inflammasome and NF-κB activity, thus indicating thymoquinone ability to act as a potential immunotherapeutic agent [145].

Intraperitoneal administration of eugenol in B6D2F1 mice bearing B16 melanoma, reduced tumor sizes, extended the mice median survival and reduced metastasis [135]. Myrtenal, one of the most abundant components in the *Teucrium polium* EO, reduced in vitro invasion and migration of both murine (B16-F0 and B16-F10) and human (SK-MEL-5) melanoma cells and metastasis induced in C57BL/6 mice bearing B16-F10 melanoma, through inhibition of the proton pump V-ATPases [143].

EOs and their main components demonstrated to affect in vitro tumor progression-associated functions and in vivo tumor growth and metastasization are reported in Table 2. The in vitro and in vivo models used are also listed in the Table.

### 3.7. Sensitization of Antitumor Agents

In addition to their ability to affect in vitro and in vivo tumor growth, several EO constituents have been reported to act synergistically with conventional chemotherapy and radiotherapy [207]. While some particular EO constituents, such as eugenol, geraniol, β-elemene, limonene, β-caryophyllene, have been shown to synergize with chemotherapy or radiotherapy in leukemia [208] or solid tumors [207,208], their efficacy in combination therapy for melanoma models are rare.

β-Elemene, one of the most active constituents of EOs from *Curcuma zedoaria* and *Curcuma wenyujin,* remarkably decreased A375 cell proliferation and enhanced apoptosis induced by radiation [209]. The effect of thymoquinone on the sensitivity of human 518A2 melanoma cells to doxorubicin was also analyzed. The authors demonstrated a cell line-dependent effect of thymoquinone on doxorubicin sensitivity inducing a synergistic proapoptotic effect on leukemia and multi-drug-resistant breast cancer cells, but not in 518A2 cells where the combination did not affect caspase kinetics or mitochondrial membrane potential but induced an antagonistic effect [208]. The authors indicate alteration in the apoptotic machinery of 518A2 cells [210] as a possible explanation of their response to thymoquinone/doxorubicin combination, thus indicating that further studies are needed to explore the effect of thymoquinone on doxorubicin sensitivity of melanoma cells. Surprisingly, the authors also reported a significant growth inhibitory effect of thymoquinone/doxorubicin combination in normal foreskin fibroblasts [208]. The ability of thymoquinone to further enhance the in vitro apoptotic effect of Gamma Knife irradiation has been reported in B16-F10, while it did not add any survival benefit to Gamma Knife treatment in C57BL/6J mice with intracerebral B16-F10 melanoma [121].

Liposomes loaded with dacarbazine and eugenol, and coated with hyaluronic acid in order to enable the active targeting of the CD44 receptor that is overexpressed by tumor cells [211], have been reported to inhibit proliferation of both human (SK-MEL-28) and mouse (B16-F10) melanoma cells and to induce both apoptosis and necrosis. The authors suggested the use of this liposome formulation to reduce dacarbazine dose during chemotherapy and consequently toxicity on normal cells [188].

EOs and their main components demonstrated to sensitize antitumor agents are reported in Table 5. The used melanoma models are also listed in the Table.

### 3.8. Chemopreventive Activity

Exposure to artificial or natural UV rays are among the major risks for the development of both non-melanoma and melanoma skin cancer. Other risk factors for melanoma development include the number of naevi and dysplastic naevi, phenotype characteristics and family history [227,228,229]. Chemopreventive agents prevent formation of cancer by multiple mechanisms, interfering with the initiation, promotion, or progression steps. Although mouse models that spontaneously develop melanoma are extremely rare, different chemically- or genetically-induced melanoma mouse models have been developed to evaluate the chemopreventive potential of compounds. Among them, the two-stage skin carcinogenesis 7,12-dimethylbenz[a]anthracene (DMBA)/12-O-tetradecanoylphobol-13-acetate (TPA) model, that fully recapitulates the multistage tumorigenesis of the skin, is the most commonly used to evaluate the chemopreventive potential of several compounds, including EOs. In particular, tumor initiation in BALB/c, CD1, ICR, SENCAR and Swiss albino models can be obtained with a single topical application of DMBA, whereas tumor promotion can be triggered by repeated applications of TPA [230]. To decrease the latency of melanoma appearance, these compounds are often administered in combination with other agents such as UV rays, or used in genetically engineered mice that harbor activating mutations in BRAF and NRAS, two oncogenes frequently mutated in human melanoma [230]. However, a drawback of DMBA/TPA models exists in the evidence that they induce the development of papillomas and small naevi more frequently than that of melanoma, making their use more accurate in the study of skin cancer on the whole, rather than of cutaneous melanoma. Thus, the chemopreventive effect of EOs or their constituents in skin cancer is discussed in this paragraph.

The chemopreventive potential of *Santalum album* EO and its major constituent α-santalol has been demonstrated in DMBA/TPA-induced skin tumors in CD1 mice: topical application of *Santalum album* EO reduced tumor incidence and multiplicity in animals. Furthermore, the chemopreventive potential of α-santalol was similar to that of *Santalum album* in DMBA/TPA-induced skin tumors in CD1 and SENCAR mice [76]. Topical application of *Salvia libanotica* EO (sage oil) delayed tumor appearance and inhibited tumor incidence and yield in DMBA/TPA in BALB/c mice, whereas decreasing EO concentration reduced only tumor yield [73]. By using the same DMBA/TPA model, *Mentha aquatica var. Kenting Water Mint* EO has been identified as a chemopreventive agent against cutaneous side effects induced by vemurafenib, a BRAF inhibitor used for treatment of melanoma patients carrying the BRAF^V600^ mutation. The results of this study evidenced that *Mentha aquatica* EO induces G2/M cell-cycle arrest and apoptosis, reduces cell viability, colony formation and the invasive and migratory functions of the mouse keratinocyte bearing HRAS^Q61L^ mutation. This mutation is found in melanoma patients with a higher probability of developing keratoacanthomas and squamous cell carcinoma after treatment with vemurafenib. In vivo treatment with *Mentha aquatica* EO decreased the formation of cutaneous papilloma and the expression of keratin14 and COX-2 observed in FVB/NJ mice exposed to DMBA/TPA and treated with vemurafenib [54].

Several studies investigated the chemopreventive potential of some components of EOs. Among them, Pal and colleagues demonstrated that oral administration of eugenol produced a reduction in the incidence and size of skin cancer in Swiss albino mice treated with DMBA and croton oil, along with an increase in the overall survival of mice. The carcinogenic process prevention by eugenol was due to reduction in cell proliferation and induction of apoptosis through the downregulation of bcl-2, c-Myc and H-ras expression along with the upregulation of active caspase 3, Bax and p53 in the skin lesions [213]. In the same year, Kaur and colleagues confirmed the inhibitory effect of eugenol in DMBA/TPA-induced skin cancer in Swiss albino mice. In particular, eugenol treatment delayed tumor onset, incidence and multiplicity when applied during the initiation, as well as during the promotion phase. The chemopreventive effect of eugenol was due to the induction of apoptosis, prevention of oxidative stress, decrease in ornitine decarboxylase activity, attenuation of tumor inflammation caused by reduction in NF-kB pathway, COX-2 and iNOS in tumor samples and in pro-inflammatory cytokines in mice serum (e.g., IL-6, TNF-α and PGE_2_) [212]. However, both groups demonstrated that mice treated with DMBA and TPA developed squamous cell carcinoma of the skin in their experimental system [212,213].

Perillyl alcohol topical treatment showed the ability to delay development and incidence of DMBA-induced melanoma in transgenic mice harboring a mutated HaRas gene driven by the tyrosinase promoter (TPras). Moreover, perillyl alcohol treatment reduced the UV-induced ROS, the levels of Ras protein and inhibited the activation of MAPK and AKT pathways in a cell line derived from DMBA-induced melanoma of TPras mice [217]. A few years later, another two studies by Chaudhary and colleagues confirmed the chemopreventive effect of perillyl alcohol and its precursor D-limonene in DMBA/TPA-induced skin cancer in Swiss albino mice. In particular, topical application of perillyl alcohol or D-limonene elicited a significant reduction in tumor incidence and tumor burden with extension of the latency period of tumor development. In agreement with Prevatt et al., perillyl alcohol or D-limonene treatment effectively reduced the skin tumorigenesis by inducing apoptosis, reducing ROS production, inflammation, Ras/Raf/ERK1/2 pathway and Bcl-2 expression along with an increase in Bax levels [177,218]. Moreover, a phase 2a clinical trial showed a modest reduction in nuclear chromatin abnormality caused by twice-daily topical application of perillyl alcohol in participants with sun-damaged skin [231]. A phase 1 clinical trial demonstrated that daily topical application of perillyl alchol cream for 30 days was well tolerated in participants with normal appearing skin [232].

The chemopreventive potential of farnesol, geraniol and menthol was analyzed by several investigators using DMBA/TPA-promoted skin tumorigenesis in Swiss albino or in ICR, a strain of albino mice. These monoterpenes reduced tumor incidence and tumor volume with an extension of latency period during the promotion phase. The mechanism of action of these three components is the same as is described for perillyl alchol, D-limonene and geraniol. In particular, their chemopreventive effects occurring through alteration of phase II detoxification agents [215], reduction in inflammation and ROS production, suppression of the Ras/Raf/ERK1/2, p38 and NF-kB signaling pathways, reduction in Bcl-2 and induction of Bax expression [177,214].

EOs and their main components demonstrated to show chemopreventive potential are reported in Table 5. The used melanoma models are also listed in the Table.

### 3.9. Antioxidant Effect

Oxidative stress producing ROS, such as superoxide, hydrogen peroxide, and hydroxyl radical, are associated with many cancer types, including non-melanoma and melanoma skin cancer, where ROS are reported to cause free radical damage to the skin [233]. Oxidative stress is involved in all stages of melanoma development, and modulates intracellular pathways related to cellular proliferation and death. Several factors, including inadequate lifestyle and/or diet or UV-irradiation lead to the formation of ROS, which are often associated with alterations in the DNA, proteins and lipids, and consequent induction of cellular aging, mutagenicity and carcinogenicity. By chelating oxidation-catalytic metals or by scavenging free radicals and ROS, natural enzymatic and non-enzymatic antioxidant defense counteracts the dangerous effects of free radicals and other oxidants. Hence the relevance of antioxidant compounds to decrease oxidative stress or damage. In this context, many plant-derived components, including EOs, are reported as a useful antioxidant source able to remove excessive free radicals and to prevent free radical-induced damage.

To evaluate the antioxidant activity of a compound of interest, several test procedures should be carried out including cell-free methods, such as 2,2′-azino-bis-3-ethylbenzthiazoline-6-sulphonic acid (ABTS) and 1,1-diphenyl-2-picrylhydrazyl (DPPH) that are the most popular and commonly employed. Other cell-free assays include hydrogen peroxide, nitric oxide, peroxynitrite radical, superoxide radical, hydroxyl radical scavenging activity, metal-ion chelating assay, lipid peroxidation and the xanthine oxidase method. Analysis of oxidation status performed in in vitro cell systems or in vivo animal models (blood or tissues) include glutathione, glutathione peroxidase, glutathione-S-transferase, superoxide dismutase and catalase activity [234].

When performing a search on PubMed, about 2800 results in the last 20 years are shown for “essential oil” and “antioxidant”. Analysis of the PubMed results identified only few research articles reporting studies on the evaluation of EO antioxidant activity in melanoma models. Among these studies, the antioxidant activity of *Melaleuca quinquenervia* EO and its constituents 1,8 cineole, α-pinene and α-terpineol [53,235], *Vetiveria zizanioides* EO and its most abundant compound, cedr-8-en-13-ol [87], *Cinnamomum cassia* EO and its major component cinnamaldehyde [32] and *Achillea Millefolium* EO with its main component linalyl acetate [23], was reflected in the recovered activities of glutathione peroxidase, superoxide dismutase, and catalase in α-MSH stimulated B16 cells. Protection from cell oxidative damage by *Lavandula Angustifolia* EO [49] and by high concentrations of *Eucalyptus camaldulensis* EO [41] was also observed in B16-F10 cells. Worthy of mention, is the in vivo study demonstrating that *Wedelia chinensis* EO (Osbeck oil) showed in vivo antioxidant activity by scavenging free radicals (lipid peroxidation and nitric oxide) and enhancing the level of endogenous antioxidants (catalase, superoxide dismutase, glutathione peroxidase, glutathione) in lung, liver and blood tissues of B16-F10-carrying C57BL/6 mice [90]. Protective effects against oxidative stress and apoptosis also in bovine aortic endothelial cells was induced by *Crocus Sativus* EO (Saffron oil) through SAPK/JNK and ERK1/2 signaling pathways, supporting its use in endothelial dysfunctions [236].

Table 6 shows studies in which the antioxidant effects of EOs and their components have been analyzed using cell-free assays or melanoma models.

### 3.10. Antimelanogenic Activity

**Figure 2:**
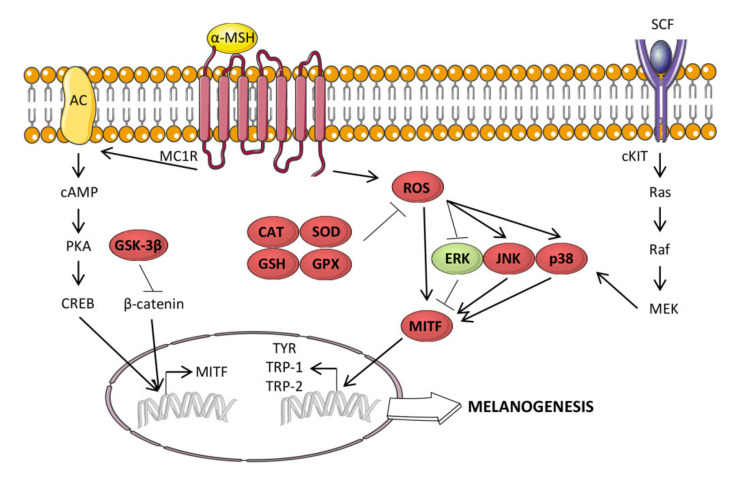
EOs and their components reduce melanogenesis and oxidation through interconnected mechanisms. Superoxide dismutase (SOD), glutathione peroxidase (GPX), catalase (CAT), glutathione (GSH), reactive oxygen species (ROS), α-melanocyte stimulating hormone (α-MSH), melanocortin 1 receptor (MC1R), adenylyl cyclase (AC), stem cell factor (SCF), microphthalmia-associated transcription factor (MITF), tyrosinase (TYR), tyrosinase-related protein -1 (TRP-1) and -2 (TRP-2), glycogen synthase kinase 3β (GSK3β), c-Jun N-terminal kinase (JNK), extracellular signal-regulated kinase (ERK). Proteins that are upregulated by EOs or their components are reported in green, proteins that are downregulated by EOs or their components are reported in red. Parts of the figure are drawn using pictures from Servier Medical Art (https://smart.servier.com).

Several EOs and their components have been reported to suppress melanogenesis, the secretion of melanin by epidermal melanocytes, and their use in skin-whitening materials has been suggested. Melanin is synthesized as a normal defense to diverse stimuli. An excessive production of melanin can be associated with hyperpigmentation and melanoma. Melanogenesis is stimulated by the melanogenic factors α-Melanocyte Stimulating Hormone (α-MSH) and Stem Cell Factor. Through the α-MSH/MC1R binding and the cyclic adenosine monophosphate-protein kinase A-cAMP response element binding protein (cAMP-PKA-CREB) signaling pathway, α-MSH stimulation activates microphthalmia-associated transcription factor (MITF), that in turn increases the expression of its target melanogenic genes, such as tyrosinase, tyrosinase-related protein-1 (TRP-1) and -2 (TRP-2). Furthermore, ERK1/2 are involved in the regulation of MITF expression through their ability to promote MITF phosphorylation and degradation [240,241]. α-MSH-induced melanogenesis is associated with ROS generation [242]. This is the reason why oxidation and melanogenesis are strictly interconnected, and EOs-induced decrease in melanin production is often attributed to EOs antioxidant property [243,244]. Figure 2 shows the effect of EOs and their components in melanogenesis and oxidation.

Several EOs, including those from *Pomelo Peel, Glechoma heredacea, Eucalyptus camaldulensis, Melaleuca quinquenervia, Chrysanthemum boreale Makino, Alpinia zerumbert, Achillea millefolium, Cinnamomum cassia, Artemisia argyi, Cryptomeria japonica* and *Vetiveria zizanioides* showed antimelanogenic activity, alongside their antioxidant properties. For these properties, they can be widely used in food, pharmaceutical, as well as in cosmetic industries as natural compounds.

The antimelanogenic properties of EO from *Pomelo Peel* have demonstrated in B16 cells where it blocked the synthesis pathway of melanin through the decrease of expression and activity of intracellular tyrosinase, without affecting cell viability and morphology [68]. Moreover, EOs from *Cryptomeria japonica* (Yakushima native cedar), *Syzygium aromaticum* (Clove oil) and *Cinnamomum zeylanicum* demonstrated antimelanogenesis activity in the same experimental model [33,81,245]. The last two EOs contained a high level of eugenol, which was also found to inhibit tyrosinase and melanogenesis in the same model [81]. An antimelanogenic characteristic of *Eucalyptus camaldulensis* EO was evidenced by its ability to inhibit the activity of mushroom tyrosinase, often used as the target enzyme in screening potential inhibitors of melanogenesis, and to decrease tyrosinase activity and intracellular melanin content of B16-F10 cells. It also reduced the expression level of MC1R, tyrosinase, TRP-1 and TRP-2, and of MAPK, JNK, PKA and ERK signaling pathways, thus suggesting the involvement of these pathways in *Eucalyptus camaldulensis EO*-mediated inhibition of melanogenesis [41]. In addition, EOs from *Psiadia terebinthina, Citrus grandis, Citrus hystrix,* and *Citrus reticulata* inhibited both intracellular and extracellular melanin production when tested against the B16-F10 model [219]. EOs from *Glechoma hederacea* [44], *Vetiveria zizanioides* [87], *Cinnamomum cassia* and its main component cinnamaldehyde [32], and *Melaleuca quinquenervia* and its main constituents, 1,8-cineole, α-pinene, and α-terpineol [53], showed potent anti-tyrosinase and antimelanogenic activities in α-MSH-stimulated B16 cells.

Other EOs showing antimelanogenic properties, in terms of reduction of melanin synthesis, intracellular tyrosinase activity and MITF expression, when tested in B16-F10 cells, include those extracted from *Vitex negundo* [88], *Origanum syriacum* and *Origanum ehrenbergii* and their main component, carvacrol [59], *Artemisia argyi* [28], *Chrysanthemum boreale* Makino [31], two varieties of *Alpinia Zerumbet* EOs, shima and tairin oils [24], *Achillea millefolium* and its component lynalyl acetate blocking melanin production through the regulation of the JNK and ERK signaling pathways [23], *Mentha aquatica* (lime mint oil) and one of its main compounds, β-caryophyllene, [221], *Vitex Trifolia* and its main component abietatriene [89].

Regarding the antimelanogenic effect of EO components, the study of Lin JH’s group reported the ability of zerumbrone to decrease melanin accumulation in B16-F10 cells, and to suppress the expression of MITF and its target genes, TYRP1 and TYRP2, after MSH stimulation with a mechanism involving ERK1/2, but not the PKA-CREB signaling pathway [226]. Antimelanogenic activity in the same melanoma model through ubiquitination and proteasomal degradation of MITF, in a ROS/ERK-dependent way, was also reported for phytol [223]. Valencene one of the major constituents of *Ocotea dispersa* EO decreased melanin content after UVB irradiation in B16-F10 cells [225]. A zebrafish embryo experimental model was also employed to demonstrate antimelanogenic activity of some EOs or their components, such as that from *Dalbergia pinnata*, which reduced tyrosinase activity and body pigmentation in zebrafish embryos [39], and thymoquinone which blocked melanogenesis also in B16-F10 mouse melanoma cells through the inhibition of the glycogen synthase kinase 3β (GSK3β)/β-catenin pathway [224]. Contrasting results were reported by the Zaidi KU group using the same experimental model and the same assays (tyrosinase activity and melanin production). The authors demonstrated that thymoquinone plays a protective role for melanogenesis [246].

Table 5 shows studies demonstrating the antimelanogenic activity of EOs and their active components in melanoma models. The used melanoma models are also listed.

## 4. Clinical Use of EOs for Cancer Patients

The side effects experienced by patients diagnosed with cancer or undergoing radio- or chemotherapy can be debilitating and can be challenging in the management of the disease. In the last few years, EOs have gained popularity as supportive therapies for cancer patients [247]. Some EOs have been reported to improve the quality of life of patients affected by cancer and showed efficacy in several side effects, such as chemotherapy-induced nausea and vomiting, mucositis, ulcer of skin, distress, depression and anxiety.

A descriptive systematic review, carried out by Boehm K et al. in 2012, highlighted short-term effects of aromatherapy, the use of EOs, on depression, anxiety, and overall wellbeing. Minimal adverse effects were reported for EO use and potential risks, including ingesting large amounts, local skin irritation, allergic contact dermatitis and phototoxicity [248]. A small feasibility study performed to evaluate the effects of mouthwash with EOs from *Leptospermum scoparium* and *Kunzea ericoides,* on mucositis of the oropharyngeal area induced by radiation during treatment for head and neck carcinoma, provided a positive effect on the development of radiation induced mucositis [249]. Topical application of the *Boswellia carterii* EO demonstrated its efficacy as supportive therapy for cancer-related fatigue in a case study [250]. Inhalation of *Citrus aurantium* EO exhibited an anxiolytic effect and reduced the symptoms associated with anxiety in patients with chronic myeloid leukaemia [251]. A cool damp washcloth with *Mentha x piperita* EO was reported to be effective in decreasing the intensity of nausea experienced by patients receiving chemotherapy [252]. Antiemetic activity of volatile oil from *Mentha spicata* and *Mentha piperita* has been also reported in chemotherapy-treated patients [253]. A recent randomized controlled trial with 120 patients evidenced the efficacy of aromatherapy with lavender and peppermint EOs in improving the sleep quality of cancer patients [254]. Linalool, linalyl acetate and menthol present in lavender or peppermint EOs [255,256] can be responsible of EOs effect on sleep due to their sedative effects. A previously performed study by M. Lisa Blackburn showed the positive effect of aromatherapy with lavender, peppermint and chamomile on insomnia and other common symptoms among 50 patients with acute leukaemia [257].

Some studies also reported the lack of effect of aromatherapy in improving sleep quality in cancer patients or in reducing chemotherapy side effects. In this regard, while non-toxic, non-invasive and well received and tolerated, the inhaled *Zingiber officinale* EO was not an effective complementary therapy for chemotherapy-induced nausea and vomiting and health-related quality of life neither in children with cancer [258] nor in women with breast cancer [259]. While not showing any harm or adverse events, the study by Sasano’s group evidenced no effects of aromatherapy on quality of life, sleep quality, and vital sign during perioperative periods of breast cancer patients [260]. Meta-analysis of three randomized controlled trials including a total of 278 participants did not show any clinical effect of aromatherapy massage on reducing pain in cancer patients [261]. Inhalation of *Lavandula angustifolia, Citrus bergamia, Cedrus atlantica* EOs administered during radiotherapy did not reduce anxiety [262]. Sample size, duration of intervention, tools used for measuring the different symptoms, use of different chemotherapies and cancer stage could account for the differences observed in the different studies.

At present, several EOs are used in clinical trials to evaluate their efficacy for symptom management in patients during cancer therapy. In order to evaluate the effect of inhaled EOs on common quality of life issues during chemotherapy, targeted therapy, and/or immunotherapy administered intravenously, a single blind, randomized controlled trial study was completed few months ago. In particular, the effect of inhalated *Zingiber officinale, German chamomile,* or *Citrus bergamia* EOs on nausea, anxiety, loss of appetite, and fatigue has been evaluated with no available information yet about the results (NCT03858855). Two clinical trials are currently open and actively recruiting patients undergoing chemotherapy to assess the perceived effectiveness to relieve symptoms of nausea or vomiting and/or anxiety, by using peppermint and lavender EOs (NCT02163369, NCT03449511). A clinical trial is actively recruiting breast cancer patients in order to test the hypothesis that an EOs blend composed of *Curcuma longa, Piper nigrum, Pelargonium asperum, Zingiber officinale, Mentha x piperita,* and *Rosmarinus officinalis*, could reduce symptoms of chemotherapy induced peripheral neuropathy, a painful, debilitating consequence of cancer treatment considered the most adverse of non-hematologic events (NCT03449303). A randomized, controlled, single blind and longitudinal study is recruiting women with breast cancer undergoing chemotherapy in order to evaluate the impact of a hedonic aroma (inhalation) on the clinical, emotional and neurocognitive variables that contribute to reduce the side effects of chemotherapy and promote quality of life (NCT03585218). A not yet recruiting clinical trial aimed at evaluating (1) the ability of peppermint and lavender aromatherapy (sniff) to promote, respectively, relief of nausea or anxiety, in an outpatient oncology setting (NCT04449315); (2) the effects of inhaled peppermint and ginger EOs, or pure vanilla extract on chemotherapy induced nausea and vomiting in men and women with breast cancer (NCT04478630) is going to start in August/September of the current year 2020.

## 5. Conclusions

Natural products have always played a pivotal role in drug discovery and in the development of many potent anticancer agents. It is, thus, desirable to continue the efforts aiming at identifying new antitumor compounds from natural sources. Based on the studies mentioned in this review, different EOs and some of their constituents appear to be suitable as a part of effective melanoma prevention, prevention of metastatic melanoma, or as complementary therapies to supplement patient care. Preclinical data also indicate the possibility of using some EO components as adjuvant agents to reduce the toxicity of drugs used in cancer prevention or therapy, such as statin [263]. Pharmacokinetic studies are needed to validate the safety and efficacy of EOs and their bioactive compounds. In fact, even if several papers indicated EOs and their components as safe and not toxic [258,259,260], hepatotoxicity described for monoterpenes and sesquiterpenes, major components of many EOs, should be also considered and should deserve more attention [264]. Further studies on terpene metabolism and toxicity need to be performed to avoid the risk of eventual liver injury. In addition, the relevance of the BRAF status of melanoma cells in response to EOs is worthy of further investigation to shed light on this issue. In fact, to the best of our knowledge, no studies have evaluated whether the antitumor effect of EOs is related with BRAF status.

Since EOs composition is affected by several factors, including geographical position and agricultural practices, an important issue to be considered is the standardization of their composition. In this context, multidisciplinary applications, including machine learning, can constitute a possible tool able to predict the bioactivity of complex mixtures and to design EOs characterized by high antineoplastic efficacy and low toxicity [93,96,265].

The studies presented in this review hold promise for further analysis of EOs as new anticancer drugs and as a source of potential anticancer supplement against melanoma. Further investigations in this area are certainly necessary, desirable, and warranted to validate the results, to ascertain the therapeutic spectrum of biological studies and to determine the clinical efficacy and safety of EOs on patients affected by melanoma.

## Figures and Tables

**Table 1:** Chemical composition of essential oils (EOs) active against melanoma models.

Plant Name from Which EOs Were Extracted	Plant Common Name	Plant Family Name	Main EO Chemical Components	Reference
*Achillea millefolium*	Yarrow, common yarrow, thousand-leaf	Asteraceae	Artemisia ketone (14.92%), camphor (11.64%), linalyl acetate (11.51%), 1,8-cineole (10.15%)	[23]
*Alpinia zerumbet*	Light Galangal, shell ginger	Zingiberaceae	γ-Terpinene (14.5%), cineole (13.8%), p-cymene (13.5%), sabinene (12.5%), terpinen-4-ol (11.9%), caryophyllene oxide (4.96%), methyl cinnamate (4.24%), caryophyllene (2.4%), γ-terpineol (1.28%)	[24]
*Annona vepretorum*	Araticum, pinha da caatinga, araticum-da-Bahia	Annonaceae	Bicyclogermacrene (35.7%), spathulenol (18.89%), α-phellandrene (8.08%), α-pinene (2.18%), o-cymene (6.24%)	[25]
*Anthemis wiedemanniana*	-	Asteraceae	9,12-Octadecadienoic acid (12.2%), hexadecanoic acid (10.5%), hexahydrofarnesyl acetone (8.3%), 1,8-cineol (6.2%), carvacrol (5.8%)	[26]
*Artemisia anomala*	-	Asteraceae or Compositae	Camphor (18.3%), 1,8-cineole (17.3%), β-caryophyllene oxide (12.7%), borneol (9.5%)	[27]
*Artemisia argyi*	-	Asteraceae or Compositae	Caryophyllene (10.19%), eucalyptol (23.66%)	[28]
*Atriplex undulata*	-	Chenopodiaceae	p-Acetanisole (28.1%), β-damascenone (9.3%), β-ionone (5.1%), viridiflorene (4.7%), 3-oxo-α-ionol (2.2%)	[29]
*Casearia lasiophylla*	-	Salicaceae	Germacrene D (18.6%), E-caryophyllene (14.7%), δ-cadinene (6.2%), α-cadinol (5.4%)	[30]
*Chrysanthemum boreale* Makino	-	Asteraceae	Germacrene D (10.6–34.9%), β-caryophyllene (10.8%), (–)-camphor (10.8–18.0%), β-thujone (11.7%), α-thujone (9.8%)	[31]
*Cinnamomum cassia*	-	Lauraceae	Cis-2-methoxycinnamic acid (43.06%), cinnamaldehyde (42.37%)	[32]
*Cinnamomum zeylanicum*	-	Lauraceae	Eugenol (70%), β-caryophyllene (2.4%)	[33]
*Citrus bergamia*	Acid lemon	Rutaceae	Limonene (38.1%), linalyl acetate (28.9%), γ-terpinene (7.3%), linalool (6.4%), β-pinene (5.4%), bergapten (1.7%)	[34]
*Citrus medica*	Citron	Rutaceae	Limonene (35.4%), γ-terpinene (24.5%), geranial (5.5%), neral (4.4%), β-pinene (2.6%), α-pinene (2.5%), β-myrcene (2.1%), terpinen-4-ol (1.5%)	[34]
*Cuminum cyminum*	Cumin-jeera	Apiaceae or Umbelliferae	Cuminaldehyde (39.48%), γ-terpinene (15.21%), O-cymene (11.82%), β-pinene (11.13%), 2-caren-10-al (7.93%), trans-carveol (4.49%), and myrtenal (3.5%)	[35]
*Curcuma aromatica*	Wild turmeric	Zingiberaceae	8,9-Dehydro-9- formyl-cycloisolongifolene (2.66–36.83%), germacrone (4.31–16.53%), ar-turmerone (2.52–17.69%), turmerone (2.62–18.38%), ermanthin (0.75–13.26%), β-sesquiphyllandrene (0.33–11.32%), ar-curcumene (0.29–10.52%)	[36]
*Curcuma kwangsiensis*	Mango-ginger	Zingiberaceae	8,9-Dehydro-9-formyl-cycloisolongifolene (2.37–42.59%), germacrone (6.53–22.20%), L-camphor (0.19–6.12%)	[37]
*Curcuma zedoaria*	Kua-zedoary	Zingiberaceae	8,9-Dehydro-9-formyl-cycloisolongifolene (60%), 6-ethenyl-4,5,6,7-tetra-hydro-3,6-dimethyl-5-isopropenyl-trans-benzofuran (12%)	[38]
*Dalbergia pinnata*	Laleng-chali	Fabaceae	Elemicin (91.06%), methyl eugenol (3.69%), 4-allyl-2,6-dimethoxyphenol (1.16%), whiskey lactone (0.55%)	[39]
*Eryngium amethystinum*	-	Apiaceae	Germacrene D (56.7%), β-elemene (4.7%), bicyclogermacrene (3.3%), α-copaene (2.2%), (E)-caryophyllene (1.9%), germacrene B (1.8%), germacra-4(15),5,10(14)-trien-1-α-ol (1.7%), cadin-4-en-10-ol (1.6%)	[40]
*Eryngium campestre*	Eryngo, field eryngo, sea-holly	Apiaceae	Germacrene D (13.8%), allo-aromadendrene (7.7%), spathulenol (7.0%), ledol (5.7%), cadin-4-en-10-ol (3.9%), γ-cadinene (3.6%), epi-α-muurolol (2.1%), germacra-4(15),5,10(14)-trien-1-α-ol (2.0%), δ-cadinene (1.9%), caryophyllene oxide (1.5%)	[40]
*Eucalyptus camaldulensis*	Murray red gum, red gum, red river gum	Myrtaceae	1,8-Cineole (23.9%), α-eudesmol (11.6%), γ-eudesmol (8.0%), and elemol (5.0%)	[41]
*Eugenia cuspidifolia*	-	Myrtaceae	Caryophyllene oxide (57.46%), α-copaene (3.75%)	[42]
*Eugenia tapacumensis*	-	Myrtaceae	Caryophyllene oxide (55.95%), α-copaene (13.67%)	[42]
*Eugenia uniflora*	Brazil cherry	Myrtaceae	Curzerene (13.4–50.6%), selina-1,3,7(11)-trien-2-one (18.1–43.1%), selina-1,3,7(11)-trien-2-one epoxidem(16.0–30.4%), germacrene B (5.0–18.4%), caryophyllene oxide(1.2–18.1%), (E)-caryophyllene (0.3–9.1%), β-elemene (3.5–8.9%), γ-elemene (2.0–7.8%)	[43]
*Glechoma hederacea*	Ground ivy, field balm, gill over the ground, runaway robin	Lamiaceae or Labiatae	Trans-3-pinanone (41.4%), 4,5,6,7-tetrahydro-5-isopropenyl-3,6-β-dimethyl-6-α-vinylbenzofuran (10.8%), β-caryophyllene (10.2%), and spathulenol (4.3%)	[44]
*Helichrysum microphyllum*	-	Asteraceae or Compositae	Neryl acetate (18.2%), rosifoliol (11.3%), δ-cadinene (8.4%), γ-cadinene (6.7%)	[45]
*Heracleum sphondylium*	Cow parsnip, eltrot	Apiaceae or Umbelliferae	Octyl acetate (54.9–60.2%), octyl butyrate (10.1–13.4%)	[46]
*Hypericum hircinum*	-	Hypericaceae	Cis-β-guaiene (29.3%), δ-selinene (11.3%), isolongifolan-7-α-ol (9.8%), (E)-caryophyllene (7.2%)	[47]
*Laurus nobilis*	Bay Tree, sweet bay, Grecian Laurel, true laurel	Lauraceae	1,8-cineole (35.15%)	[48]
*Lavandula augustifolia*	English lavender, true lavender	Lamiaceae or Labiatae	α-Pipene, β-pipene, camphene, eucalyptol, D-limonene	[49]
*Lippia gracilis*	-	Verbenaceae	Thymol (55.50%), p-cymene (10.80%), γ-terpinene (5.53%), myrcene (4.03%)	[50]
*Liriodendron tulipifera*	Tulip tree, tulip poplar, yellow poplar, canary whitewood	Magnoliaceae	(*Z*)-β-Ocimene (12.5–25.2%), (E)-β-ocimene (3.7–6.8%), β-elemene (16.4–17.1%), germacrene D (18.9–27.2%)	[51]
*Melaleuca alternifolia*	Tea Tree	Myrtaceae	Terpinen-4-ol (42.35%), γ-terpinene (20.65%), α-terpinene (9.76%)	[52]
*Melaleuca quinquenervia*	-	Myrtaceae	1,8-Cineole (21.06%), α-pinene (15.93%), viridiflorol (14.55%), α-terpineol (13.73%)	[53]
*Mentha aquatica*	-	Lamiaceae or Labiatae	β-Ocimene (22.18%), β-pinene (15.41%), 1,8-cineole (12.87%), α-pinene (10.49%)	[54]
*Myrcia laruotteana*	-	Myrtaceae	α-Bisabolol (23.6%), α-bisabolol oxide B (11.5%)	[55]
*Myristica fragrans*	Mace, nutmeg	Myristicaceae	Myristicin, limonene, eugenol and terpinen-4-ol	[56]
*Nectandra leucantha*	-	Lauraceae	Bicyclogermacrene (28.44%), germacrene A (7.34%)	[57]
*Ocimum basilicum*	Sweet basil, common basil, thai basil, tropical basil	Lamiaceae or Labiatae	1,8 Cineole (11.0%), linalool (42.5%), estragole (33.1%)	[58]
*Ocimum gratissimum*	African basil, east Indian basil, russian basil, shrubby basil	Lamiaceae or Labiatae	Eugenol (54.0%), 1,8 cineole (21.6%), β-selinene (5.5%), β-caryophyllene (5.3%), (Z)-ocimene (4.0%)	[58]
*Ocimum micranthum*	-	Lamiaceae or Labiatae	Eugenol (64.8%), β-caryophyllene (14,3%), bicyclogermacrene (8.1%)	[58]
*Ocimum tenuiflorum*	Sacred basil	Lamiaceae or Labiatae	Eugenol (59.4%), β-caryophyllene (29.4%), germacrene A (8.1%)	[58]
*Origanum ehrenbergii*	-	Lamiaceae or Labiatae	Carvacrol, thymoquinone	[59]
*Origanum syriacum*	Bible hyssop	Lamiaceae or Labiatae	Carvacrol, thymoquinone	[59]
*Perilla frutescens*	Shiso, beefsteakplant, spreading beefsteak plant	Lamiaceae or Labiatae	isoegomaketone	[60]
*Piper aleyreanum*	-	Piperaceae	β-Elemene (16.3%), bicyclogermacrene (9.2%), δ-elemene (8.2%), germacrene D (6.9%), β-caryophyllene (6.2%), spathulenol (5.2%)	[61]
*Piper cernuum*	-	Piperaceae	α-Pinene, camphene, limonene, carvacrol, tymol, myrcene, p-cymene, aterpineol, linalol	[62]
*Piper klotzschianum*	-	Piperaceae	Germacrene D (7.3–22.8%), bicyclogermacrene (13.4–21.6%), E)-caryophyllene (11.9–16.8%), β-pinene (2.3–27.2%), α-pinene (1.4–7.2%)	[63]
*Pistacia lentiscus*	Chios mastictree, aroeira, lentiscus, lentisk, mastic, mastictree	Anacardiaceae	Perillyl alcohol	[64]
*Pituranthos tortuosus*	-	Apiaceae	Sabinene (24.24%), α-pinene (17.98%), limonene (16.12%), and terpinen-4-ol (7.21%)	[65,66]
*Plectranthus amboinicus*	Country borage, Indian borage	Lamiaceae or Labiatae	Carvacrol thymol, cis-caryophyllene, trans-caryophyllene, and p-cymene	[67]
*Pomelo peel*	-	Rutaceae	Limonene (55.92%), β-myrcene (31.17%), β-pinene (3.16%), ocimene (1.42%), β-copaene (1.24%)	[68]
*Porcelia macrocarpa*	-	Annonaceae	Germacrene D (47%), bicyclogermacrene (37%), verbanyl acetate (0.5%), phytol (1.2%)	[69]
*Pterodon emarginatus*	Faveiro, sucupira, sucupira-branca	Fabaceae	β-Elemene (15.3%), trans-caryophyllene (35.9%), α-humulene (6.8%), germacrene-D (9.8%), bicyclogermacrene (5.5%), spathulenol (5.9%)	[70]
*Salvia aurea*	-	Lamiaceae or Labiatae	Caryophyllene oxide (12.5%), α-amorphene (12.0%), aristolone (11.4%), aro-madendrene (10.7%), elemenone (6.0%)	[71]
*Salvia breacteata*	-	Lamiaceae or Labiatae	Caryophyllene oxide (16.6%)	[72]
*Salvia judaica*	-	Lamiaceae or Labiatae	Caryophyllene oxide (12.8%)	[71]
*Salvia libanotica*	-	Lamiaceae or Labiatae	Cineole (57.4%), camphor (8.4%), β-pinene (5.1%), α-pinene (3.9%), camphene (3.0%)	[73]
*Salvia officinalis*	Sage, kitchen sage, small leaf sage, garden sage	Lamiaceae or Labiatae	Caryophyllene (25.634%), camphene (14.139%), eucalyptol (13.902%)	[74]
*Salvia rubifolia*	-	Lamiaceae or Labiatae	γ-Muurolene (11.8%)	[72]
*Salvia verbenaca*	Wild clary	Lamiaceae or Labiatae	Hexadecanoic acid (11–23.1%), Z)-9-octadecenoic acid (5.6–11.1%), benzaldehyde (1.1–7.3%)	[75]
*Salvia viscosa*	-	Lamiaceae or Labiatae	Caryophyllene oxide (12.7%)	[71]
*Santalum album*	White sandal tree, sandalwood, sandal tree, sandal	Santalaceae	α-Santalol (61%), β-santalol (28%)	[76]
*Satureja hortensis*	Summer savory	Lamiaceae or Labiatae	γ-Terpinene (37.862%), o-cymene (15.113%), thymol (13.491%), carvacrol (13.225%)	[77]
*Schinus terebinthifolius* Raddi	Brazilian pepper tree	Anacardiaceae	β-Longipinene (8.1%), germacrene D (23.8%), biclyclogermacrene (15.0%), α-pinene (5.7%), β-pinene (9.1%)	[78]
*Stachys germanica*	Downy woundwort, German hedgenettle	Lamiaceae or Labiatae	(Z,Z,Z)-9,12,15-octa-decatrienoic acid methyl ester (33.3%), exadecanoic acid (22.1%)	[79]
*Stachys parviflora*	-	Lamiaceae or Labiatae	α-Terpenyl acetate (23.6%), β-caryophyllene (16.8%), bicyclogermacrene (9.3%), spathulenol (4.9%), α-pinene (4.2%)	[80]
*Syzygium aromaticum*	Clove, Zanzibar redhead	Myrtaceae	Eugenol (61%), (β-carophillene 5.7%)	[33,81]
*Tagetes erecta*	African marigold, Aztec marigold, big marigold, American marigold	Asteraceae or Compositae	Limonene (10.4%), α-terpinolene (18.1%), (E)-ocimenone (13.0%), dihydrotagetone (11.8%)	[82]
*Tanacetum macrophyllum*	Tansy, rayed tansy, tansy chrysanthemum	Asteraceae or Compositae	Germacrene D (6.9–30.9%), 10-epi-γ-eudesmol (3.9–13.5%), camphor (11.1%), linalool (0.6–10.8%), 1,8-cineole (5.5–8.8%)	[83]
*Thymus alternans*	-	Lamiaceae or Labiatae	(E)-Nerolidol (15.8–31.4%), germacrene D (6.7–7.4%), geranial (6.8–7.7%), (E)-β-ocimene (2.6–7.0%), linalool (1.7–6.4%), geraniol (3.3–6.2%), neral (4.9–5.4%)	[84]
*Thymus munbyanus*	-	Lamiaceae or Labiatae	Borneol (31.2–44.8%), camphor (5.7–13.6%), camphene (3.6–7.5%), 1,8-cineole (4.2–6.0%), germacrene D (3.1–5.0%)	[85]
*Thymus vulgaris*	English thyme, French thyme, garden thyme, thyme	Lamiaceae or Labiatae	γ-Terpinene (68.415%), thymol (24.721%), caryophyllene (5.5%), α-pinene (4.816%)	[74]
*Tridax procumbens*	Coat buttons, coat-button, Mexican daisy	Asteraceae or Compositae	α-Pipene, β-pinene, phellandrene, sabinene	[86]
*Vetiveria zizanioides*	Cuscus grass, khus-khus, khas-khas, vetiver	Poaceae	Cedr-8-en-13-ol (12.4%), α-amorphene (7.80%), β-vatirenene (5.94%), α-gurjunene (5.91%), dehydroaromadendrene (5.45%)	[87]
*Vitex Negundo*	Common chaste tree, negundo, five keaved chaste tree, negundo chastetree, chaste tree	Lamiaceae or Labiatae	Sabinene (19.04%), caryophyllene (18.27%)	[88]
*Vitex Trifolia*	Indian privet, Arabian lilac, Indian three-leaf vitex, hand of mary	Lamiaceae or Labiatae	α-Pinene (11.38%), β-pinene (2.84%), sabinene (10.25%), eucaluptol (8.60%), camphene (12.69%), manoyl oxide (16.11%), abietatriene (9.03%)	[89]
*Wedelia chinensis*	Chinese wedelia	Asteraceae or Compositae	Carvocrol, trans-caryophyllene	[90]
*Zornia brasiliensis*	-	Fabaceae	Trans-nerolidol (48.0%), germacrene D (13.9%), α-humulene (9.3%), trans-caryophyllene (8.4%), and (Z,E)-α-farnesene (7.3%)	[91]

where not indicated, some plant common names and EOs composition were not available from the cited paper. EOs from *Boswellia carterii, Citrus grandis, Citrus hystix, Citrus reticulate, Psiadia terebinthina* were not included in the table as their chemical compositions were not reported in the corresponding cited articles.

**Table 2:** EOs and their components that have been demonstrated to affect in vitro or in vivo melanoma growth and metastasization.

Pathway Affected	Plant Name from Which EOs Were Extracted	EO Active Components	In Vitro and In Vivo Models	Reference
In vitro cell proliferation	*Annona Vepretorum*	Spathulenol, o-cymene, α-pinene	B16-F10	[25]
	*Anthemis wiedemanniana*	-	C32	[26]
	*Artemisia anomala*	-	BRO	[27]
	*Casearia lasiophylla*	-	UACC-62	[30]
	*Citrus bergamia*	Bergapten	A375	[34]
	*Citrus medica*	Limonene	A375	[34,126]
	*Coleus aromaticus*	Carvacrol	A375	[127]
	*Curcuma aromatica*	-	B16	[36]
	*Curcuma kwangsiensis*	-	B16	[37]
	*Curcuma zedoaria*	-	B16-Bl6	[38]
	*Eryngium amethystinum*	-	A375	[40]
	*Eryngium campestre*	-	A375	[40]
	*Eugenia cuspidifolia*	-	SK-MEL-19	[42]
	*Eugenia tapacumensis*	-	SK-MEL-19	[42]
	*Eugenia uniflora*	Curzerene	SK-MEL-19	[43]
	*Helichrysum microphyllum*	-	A375	[45]
	*Heracleum sphondylium*	Octyl butyrate	A375	[46]
	*Hypericum hircinum*	-	B16-F1	[47]
	*Laurus nobilis*	-	C32	[48]
	*Lippia gracilis*	-	B16-F10	[50,128]
	*Liriodendron tulipifera*	β-Elemene	A375	[51]
	*Melaleuca alternifolia*	Terpinen-4-ol	A375, M14, B16-F10	[52,129,130]
	*Melaleuca quinquenervia*	1,8-Cineole, α-Pipene, α-Terpineol	B16	[53]
	*Myrcia laruotteana*	-	UACC-62	[55]
	*Nectandra leucantha*	Bicyclogermacrene	B16-F10-Nex2	[57]
	*Perilla frutescens*	Isoegomaketone	B16	[60]
	*Piper aleyreanum*	-	SK-MEL-19	[61]
	*Piper cernuum*	Camphene	B16-F10-Nex2	[62]
	*Piper klotzschianum*	-	B16-F10	[63]
	*Porcelia macrocarpa*	-	B16-F10-Nex2	[69]
	*Pterodon emarginatus*	-	MeWo	[70]
	*Salvia aurea*	-	M14, A375, A2058	[71]
	*Salvia bracteata*	-	M14	[72]
	*Salvia judaica*	-	M14, A375, A2058	[71]
	*Salvia officinalis*	-	A375, M14, A2058, B164A5	[74,101]
	*Salvia rubifolia*	-	M14	[72]
	*Salvia verbenaca*	-	M14	[75]
	*Salvia viscosa*	-	M14, A375, A2058	[71]
	*Satureja hortensis*	-	B164A5, A375	[77]
	*Schinus terebinthifolius* Raddi	α-Pipene, β-pipene, pipane	B16-F10-Nex2, A2058	[78]
	*Stachys germanica*	-	C32	[79]
	*Stachys parviflora*	-	B16-F10	[80]
	*Syzygium aromaticum*	Eugenol	B16	[81]
	*Tagetes erecta*	-	B16-F10	[82]
	*Tanacetum macrophyllum*	-	A375	[83]
	*Thuja occidentalis*	Thujone	A375	[131]
	*Thymus alternans*	-	A375	[84]
	*Thymus munbyanus*	-	A375	[85]
	*Thymus vulgaris*	-	B164A5, A375	[74]
	*Vitex Trifolia*	Abietatriene	B16-F10	[89]
		Carvacrol	SK-MEL-2	[132]
		Citral	B16-F10, SK-MEL-147, UACC-257	[133]
		Eugenol	SK-MEL-2, A2058, SK-MEL-28, Sbcl2, WM3211, WM98-1, WM1205Lu, LCM-MEL GR-MEL, 13443	[132,134,135,136]
		Farnesol	B16, B16-F10	[106,109]
		Farnesyl anthranilate	B16	[106,107]
		Farnesyl-O-acetylhydroquinone	B16	[106]
		Menthol	A375	[137]
		Neridol	B16	[109]
		Thymol	SK-MEL-2	[132]
		Zerumbone	CHL-1, A375	[138,139]
		β-Caryophyllene	B16-F10	[140]
In vitro tumor progression-associated functions	*Alpinia zerumbet*	-	HUVEC	[141]
	*Eugenia uniflora*	Curzerene	SK-MEL-19	[43]
	*Melaleuca alternifolia*	Terpinen-4-Ol	M14	[142]
	*Pituranthos tortuosus*	-	B16-F10	[66]
	*Satureja hortensis*	-	B164A5, A375	[77]
		Myrtenal	B16-F0, B16-F10, SK-MEL-5	[143]
		Thujone	B16-F10	[144]
		Thymoquinone	B16-F10, A375	[145]
		Zerumbone	CHL-1	[138]
In vivo tumor growth and metastasization	*Annona Vepretorum*	Spathulenol, o-cymene, α-pinene	B16-F10 (C57BL/6J)	[25]
	*Boswellia carterii*	-	B16-F10 (C57BL/6)	[146]
	*Curcuma zedoaria*	-	B16-Bl6 (C57BL/6)	[38]
	*Melaleuca alternifolia*	-	B16-F10 (C57BL/6J)	[147]
	*Perilla frutescens*	Isoegomaketone	B16 (C57BL/6N)	[60]
	*Piper cernuum*	Camphene	B16-F10-Nex2 (C57BL/6)	[62]
	*Pituranthos tortuosus*	-	B16-F10 (BALB/c)	[65,66]
	*Plectranthus amboinicus*	-	B16-F10 (C57BL/6)	[67]
	*Salvia officinalis*	β-Ursolic acid	B16 (C57BL/6)	[148]
	*Schinus terebinthifolius* Raddi	α-Pipene	B16-F10-Nex2 (C57BL/6)	[149]
	*Tridax procumbens*	-	B16-F10 (C57BL/6)	[86]
	*Zornia brasiliensis*	-	B16-F10 (C57BL/6)	[91]
		Eugenol	B16 (B6D2F1)	[135]
		Limonene	B16-F10 (C57BL/6)	[150]
		Myrtenal	B16-F10 (C57BL/6)	[143]
		Perillic Acid	B16-F10 (C57BL/6)	[150]
		Thujone	B16-F10 (C57BL/6)	[144]
		Thymoquinone	B16-F10 (C57BL/6)	[121,151]
		α-Pinene	B16-F10-Nex2 (C57BL/6)	[149]
		β-Caryophyllene	B16-F10 (C57BL/6N)	[140]
		β-Elemene	B16-F10 (C57BL/6)	[120,152]

where not indicated, EO active components were not available from the cited articles. When referred to in vivo studies, the murine strain used is indicated in brackets. Human Umbilical Vein Endothelial Cells (HUVEC).

**Table 3:** EOs and their components that were demonstrated to affect cell cycle distribution, apoptosis, necrosis and autophagy of melanoma cells.

Pathway Affected	Plant Name from Which EOs Were Extracted	EO Active Components	In Vitro and In Vivo Melanoma Models	Reference
Cell cycle	*Melaleuca alternifolia*	Terpinen-4-ol	A375, B16	[129,130]
	*Santalum album*	α-Santol	UACC-62	[155]
		Eugenol	A2058, WM1205Lu, Sbcl2, WM3211	[134,135]
		Farnesol	B16	[109]
		Neridol	B16	[109]
Apoptosis	*Annona Vepretorum*	Spathulenol, o-cymene, α-pinene	B16-F10	[25]
	*Boswellia carterii*		B16-F10, FM94	[146]
	*Coleus aromaticus*	Carvacrol	A375	[127]
	*Eugenia uniflora*	Curzerene	SK-MEL-19	[43]
	*Melaleuca alternifolia*	Terpinen-4-Ol	A375, M14	[52,129]
	*Perilla frutescens*	Isoegomaketone	B16	[60]
	*Piper cernuum*	Camphene	B16-F10-Nex2	[62]
	*Pituranthos tortuosus*	-	B16-F10	[66]
	*Salvia aurea*	-	M14	[71]
	*Salvia bracteata*	-	M14	[72]
	*Salvia judaica*	-	M14	[71]
	*Salvia officinalis*	-	A375, M14, A2058	[101]
	*Salvia verbenaca*	-	M14	[75]
	*Salvia viscosa*	-	M14	[71]
	*Salvia rubifolia*	-	M14	[72]
	*Schinus terebinthifolius* Raddi	α-Pipene	B16-F10-Nex2, A2058	[78,149]
	*Thuja occidentalis*	Thujone	A375	[131]
	*Tridax procumbens*	-	B16-F10 (C57BL/6)	[86]
		Citral	B16-F10, SK-MEL-147, UACC-257	[133]
		Eugenol	A2058, SK-MEL-28	[134,135,136]
		Linalool	RPMI-7932	[160]
		Menthol	A375, G-361	[161,162]
		Thymoquinone	B16-F10	[121]
		Zerumbone	CHL-1, A375	[138,139]
		β-Carophyllene	B16-F10 (C57BL/6N)	[140]
Necrosis and autophagy	*Melaleuca alternifolia*	Terpinen-4-Ol	B16	[130]
	*Salvia aurea*	-	M14	[71]
	*Salvia bracteata*	-	M14	[72]
	*Salvia judaica*	-	M14	[71]
	*Salvia viscosa*	-	M14	[71]
	*Salvia rubifolia*	-	M14	[72]
		Citral	B16-F10	[133]

where not indicated, EO active components were not available from the cited articles. When referred to in vivo studies, the murine strain used is indicated in brackets.

**Table 4:** EOs and their components that have been demonstrated to affect in vitro and in vivo angiogenesis and lymphangiogenesis.

Plant Name from Which EOs Were Extracted	EO Active Components	In Vitro and In Vivo Models	Reference
*Citrus lemon*	-	CAM	[189]
*Curcuma zedoaria*	-	HUVEC, CAM, rat aortic ring assay, B16-Bl6 (C57BL/6)	[38]
*Hypericum perforatum*	-	EAhy.926	[187]
*Myristica fragrans*	-	EAhy.926	[56]
*Pistacia lentiscus*	-	B16	[64]
*Plectranthus amboinicus*	-	B16-F10 (C57BL/6)	[67]
*Salvia officinalis*	-	Infected wound model (BALB/c)	[190]
*Tridax procumbens*	-	B16-F10 (C57BL/6)	[86]
	Curcumol	HUVEC	[199]
	Eugenol	EAhy.926	[188]
	Perillyl Alcohol	BLMVEC, HUVEC, B16-F10	[198]
	Zerumbone	HUVEC, CAM, rat aortic ring assay	[195,196]
	β-Caryophyllene	B16-F10 (C57BL/6N)	[140]
	β-Elemene	CAM, rat aortic ring assay, B16-F10 (C57BL/6)	[120,152]

Human Umbilical Vein Endothelial Cells (HUVEC), transformed human umbilical vein endothelial cells produced by fusion of A549/8 lung adenocarcinoma with human umbilical endothelial cells (EAhy.926), Bovine Lung Microvascular Endothelial Cells (BLMVEC), chick embryo chorioallantoic membrane (CAM). Where not reported, EO active components were not available in the cited paper. When referrring to in vivo studies, the murine strain used is indicated in brackets.

**Table 5:** Effect of EOs and their components in the sensitization of antitumor agents, chemoprevention and melanogenesis.

Pathway Affected	Plant Name from Which EOs Were Extracted	EO Active Components	In Vitro and In Vivo Models	Reference
Sensitization of antitumor agents		Eugenol	SK-MEL-28, B16-F10	[188]
		Thymoquinone	B16-F10	[121]
		β-Elemene	A375	[209]
Chemoprevention	*Mentha aquatica*	-	DMBA/TPA (FVB/NJ)	[54]
	*Salvia libanotica*	-	DMBA/TPA (BALB/c)	[73]
	*Santalum album*	α-Santol	DMBA/TPA (CD1, SENCAR)	[76]
		Eugenol	DMBA/TPA, DMBA/croton oil (Swiss albino)	[212,213]
		Farnesol	DMBA/TPA (Swiss albino)	[214]
		Geraniol	DMBA/TPA (Swiss albino)	[215]
		Limonene	DMBA/TPA (Swiss albino)	[177]
		Menthol	DMBA/TPA (ICR)	[216]
		Perillyl Alcohol	DMBA/TPras mut, DMBA/TPA (Swiss albino)	[217,218]
Melanogenesis	*Achillea millefolium*	Linalyl Acetate	B16	[23]
	*Alpinia zerumbet*	-	B16-F10	[24]
	*Artemisia argyi*	-	B16-F10	[28]
	*Cinnamomum cassia*	Cinnamaldehyde	B16	[32]
	*Cinnamomum zeylanicum*	-	B16	[33]
	*Citrus grandis*	-	B16-F10	[219]
	*Citrus hystrix*	-	B16-F10	[219]
	*Citrus reticulata*	-	B16-F10	[219]
	*Cryptomeria japonica*	-	B16	[220]
	*Chrysanthemum boreale* Makino	Cuminaldehyde	B16-Bl6	[31]
	*Dalbergia pinnata*	-	Zebrafish embryos	[39]
	*Eucalyptus camaldulensis*	-	B16-F10	[41]
	*Glechoma hederacea*	-	B16	[44]
	*Melaleuca quinquenervia*	1,8-Cineole, α-pipene, α-terpineol	B16	[53]
	*Mentha aquatica*	β-Caryophyllene	B16-F10	[221]
	*Origanum syriacum*	Carvacrol	B16-F1	[59,222]
	*Origanum ehrenbergii*	Carvacrol	B16-F1	[59]
	*Pomelo peel*	-	B16	[68]
	*Psiadia terebinthina*	-	B16-F10	[219]
	*Syzygium aromaticum*	Eugenol	B16	[81]
	*Vetiveria zizanioides*	Cedr-8-En-13-Ol	B16	[87]
	*Vitex Negundo*	-	B16-F10	[88]
	*Vitex Trifolia*	Abietatriene	B16-F10	[89]
		Phytol	B16-F10	[223]
		Thymoquinone	B16-F10	[224]
		Valencene	B16-F10	[225]
		Zerumbone	B16-F10	[226]

where not indicated, EO active components were not available from the cited articles. When referred to in vivo studies, the murine strain used is indicated in brackets. 7,12-dimethylbenz[a]anthracene (DMBA), 12-O-tetradecanoylphobol-13-acetate (TPA), HaRas gene driven by the tyrosinase promoter (TPras).

**Table 6:** EOs and their components showing antioxidant effect in cell-free assay or in melanoma models.

Plant Name from Which EOs Were Extracted	EO Active Components	Cell Free Assay and Melanoma Models	Reference
*Achillea millefolium*	Linalyl Acetate	B16	[23]
*Alpinia zerumbet*	-	DPPH, ABTS, nitric oxide, hydroxyl radical scavenging activity, xanthine oxidase	[24]
*Artemisia argyi*	-	DPPH, ABTS, metal-ion chelation	[28]
*Atriplex undulata*	-	Crocin bleaching inhibition, DPPH	[29]
*Cinnamomum cassia*	Cinnamaldehyde	B16	[32]
*Chrysanthemum boreale Makino*	-	DPPH, ABTS	[31]
*Cryptomeria japonica*	-	B16	[220]
*Cuminum Cyminum*	-	DPPH, superoxide anion radical-scavenging activity, β-carotene/linoleic acid	[35]
*Curcuma aromatica*	-	DPPH	[36]
*Curcuma kwangsiensis*	-	DPPH	[37]
*Dalbergia pinnata*	-	DPPH, ABTS	[39]
*Eucalyptus camaldulensis*	-	DPPH, ABTS, B16-F10	[41]
*Eugenia uniflora*	-	DPPH, β-carotene/linoleic acid	[43]
*Glechoma hederacea*	-	β-carotene/linoleic acid, B16	[44]
*Helichrysum microphyllum*	-	DPPH, ABTS	[45]
*Hypericum hircinum*	-	DPPH, ABTS	[47]
*Lavandula augustifolia*	-	B16-F10	[49]
*Melaleuca quinquenervia*	1,8-Cineole, α-pipene, α-terpineol	B16	[53]
*Ocimum basilicum*	-	DPPH, hypoxanthine/xanthine oxidase	[58]
*Ocimum gratissimum*	-	DPPH, hypoxanthine/xanthine oxidase	[58,237]
*Ocimum micranthum*	-	DPPH, hypoxanthine/xanthine oxidase	[58]
*Ocimum tenuiflorum*	-	DPPH, hypoxanthine/xanthine oxidase	[58]
*Piper aleyreanum*	-	DPPH	[61]
*Psidium guineense*	-	DPPH, ABTS	[238]
*Pomelo peel*	-	DPPH, ABTS	[68]
*Satureja hortensis*	-	DPPH	[77]
*Stachys cretica*	-	DPPH	[79]
*Stachys hydrophila*	-	DPPH	[79]
*Stachys palustri*	-	DPPH	[79]
*Stachys parviflora*	-	DPPH, β-carotene/linoleic acid	[80]
*Tanacetum macrophyllum*	-	DPPH, ABTS, FRAP	[83]
*Thymus munbyanus*	-	DPPH, ABTS, FRAP	[85]
*Thymus vulgaris*	-	DPPH	[125]
*Vetiveria zizanioides*	-	β-carotene/linoleic acid, B16	[87]
*Vitex Negundo*	-	DPPH, ABTS, metal-ion chelation	[88]
*Wedelia chinensis*	-	B16-F10 (C57BL/6)	[90,239]
	Eugenol	A2058, SK-MEL 28	[134]

where not indicated, EO active components were not available from the cited articles. When referred to in vivo studies, the murine strain used is indicated in brackets. 1,1-diphenyl-2-picrylhydrazyl (DPPH), 2,2′-azino-bis-3-ethylbenzthiazoline-6-sulphonic acid (ABTS), ferric reducing/antioxidant power (FRAP).
